# Comparative single‐cell transcriptomic profiling of patient‐derived renal carcinoma cells in cellular and animal models of kidney cancer

**DOI:** 10.1002/2211-5463.70022

**Published:** 2025-04-16

**Authors:** Richard Huang, Lynn Kee, Alexander Gont, Jalna Meens, Fraser G. Ferens, Meredith S. Irwin, Laurie Ailles, Scott A. Yuzwa, Claire M. Robinson, Michael Ohh

**Affiliations:** ^1^ Department of Laboratory Medicine and Pathobiology University of Toronto Canada; ^2^ Cell Biology Program The Hospital for Sick Children Toronto Canada; ^3^ Princess Margaret Cancer Centre University Health Network Toronto Canada; ^4^ Department of Medical Biophysics University of Toronto Canada; ^5^ Department of Paediatrics The Hospital for Sick Children Toronto Canada; ^6^ School of Medicine, Health Sciences Centre University College Dublin Dublin 4 Ireland; ^7^ Conway Institute of Biomolecular and Biomedical Research University College Dublin Dublin 4 Ireland; ^8^ Department of Biochemistry University of Toronto Canada

**Keywords:** animal models of carcinogenesis, clear cell renal cell carcinoma, *In vitro* models of carcinogenesis, kidney cancer, single‐cell RNA‐sequencing

## Abstract

Clear cell renal cell carcinoma (ccRCC) is the most common form of kidney cancer that often displays resistance to conventional cancer therapies, including chemotherapy and radiation therapy. Targeted treatments, including immunotherapies and small molecular inhibitors, have been associated with improved outcomes. However, variations in the patient response and the development of resistance suggest that more models that better recapitulate the pathogenesis and metastatic mechanisms of ccRCC are required to improve our understanding and disease management. Here, we examined the transcriptional landscapes of *in vitro* cell culture as well as *in vivo* orthotopic and metastatic NOD/SCID‐γ mouse models of ccRCC using a single patient‐derived RCC243 cell line to allow unambiguous comparison between models. In our mouse model assays, RCC243 cells formed metastatic tumors, and all tumors retained clear cell morphology irrespective of model type. Notably, gene expression profiles differed markedly between the RCC243 tumor models—cell culture, orthotopic tumors, and metastatic tumors—suggesting an impact of the experimental model system and whether the tumor was orthotopic or metastatic. Furthermore, we found conserved prognostic markers between RCC243 tumor models and human ccRCC patient datasets, and genes upregulated in metastatic RCC243 were associated with worse patient outcomes.

## Graphical abstract

We generated and characterized clear cell renal cell carcinoma models using the patient‐derived RCC243 cell line—including cell culture, orthotopic, and metastatic tumors—via single‐cell RNA‐sequencing for comparisons between models and patient tumor datasets. RCC243 models were representative of human ccRCC that can be used to investigate kidney cancer progression and showed that the model system used impacts the gene expression profile.

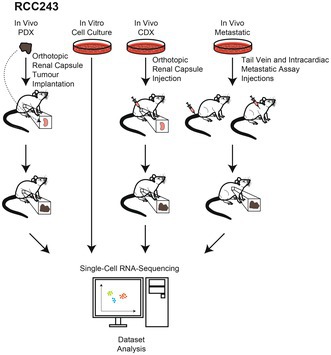

**Abbreviations**
ccRCC: clear cell renal cell carcinoma
CDX: cell‐line‐derived xenograft
EGA: European Genome‐Phenome Archive
GEMMs: genetically engineered mouse models
GEO: Gene Expression Omnibus
GSEA: gene set enrichment analysis
H&E: hematoxylin and eosin
HIF: hypoxia‐inducible factor
PDX: patient‐derived xenograft
VHL: von Hippel–Lindau

Kidney cancer is one of the ten most commonly diagnosed malignancies, with increasing incidence around the world [1, 2]. Renal cell carcinoma (RCC) accounts for approximately 90% of kidney cancer, and clear cell renal cell carcinoma (ccRCC) is the most common RCC histologic subtype [3, 4, 5]. Most patients with ccRCC present with sporadic tumors that are commonly characterized by the loss or inactivation of the von Hippel–Lindau (VHL) tumor suppressor gene [6]. Individuals with the inherited cancer predisposition, VHL syndrome, are at an elevated risk due to the inheritance of a mutated *VHL* allele [7]. The subsequent loss of heterozygosity event that occurs in both patients with sporadic disease and VHL disease results in the dysregulation of the hypoxia response pathway that can upregulate genes associated with metabolism, angiogenesis, cellular migration, and cell survival, which is consistent with the highly vascular tumors and clear cell phenotype observed in ccRCC [8, 9, 10]. This includes the stabilization of HIF‐2α, which has been characterized as a driver for ccRCC pathogenesis [11, 12]. Other driver mutations affecting tumor suppressor genes, such as *PBRM1*, *BAP1*, and *SETD2*, have also been characterized with variations in their inactivation status, highlighting the significant amount of intratumoral heterogeneity within a patient as well as heterogeneity between patients, which can have implications on patient responses to therapy [3, 6, 13, 14]. In addition, mechanisms involving epigenetic remodeling, transcriptional enhancers, and cellular inflammatory responses have been implicated in the development of metastatic ccRCC [15, 16, 17].

There have been many recent advances in the treatment of ccRCC, including small molecular inhibitors and immunotherapies that have led to improved patient outcomes [3, 18, 19]. However, despite these advances, patients who present with distant metastatic disease, most often in the lungs, have five‐year survival rates below 20% [1, 20]. In addition, patients with localized disease who were previously treated with surgical resection can often relapse with metastatic disease, which underscores the need for a better understanding of the genes and pathways regulating metastatic spread [3].

Cellular and animal models for ccRCC have been essential to the discovery of mechanisms involved in tumorigenesis and metastasis, as well as the identification and pre‐clinical testing of novel therapies [12, 21]. These include cell lines and *in vivo* mouse models such as cell‐line‐derived xenograft (CDX), patient‐derived xenograft (PDX), and genetically engineered mouse models (GEMMs) [10, 21, 22]. However, there are caveats to the use of cell lines and *in vivo* models. Commercially available cell lines, including A‐498, contain copy number amplifications and deletions that are not characteristic of ccRCC patient tumors, and some cell lines such as 786‐O do not recapitulate a prominent clear cell histology in CDX models, which is in contrast to cell lines derived from patient tumors with genetic profiles more similar to human tumors [10, 15, 17, 21, 23, 24]. The cell line models have also often been used to generate metastatic cell lines through tail vein and orthotopic injections to enrich for metastatic clones, which has allowed for an improved understanding of metastatic markers [15, 17]. PDX models are considered the gold standard to understand tumor heterogeneity and perform pre‐clinical drug testing [10, 22]. However, these models require the use of immunocompromised mice such as the NOD/SCID‐γ, which do not enable characterization of the role of the immune system or microenvironment in ccRCC pathogenesis [21]. Instead, GEMMs have allowed for the investigation of spontaneous ccRCC development and patient therapies within an immunocompetent setting, but the simultaneous mutations involving *Vhl* with *Pbrm1* or *Bap1* in these models or the lack of dependence on HIF‐2 for tumorigenesis do not represent the typical developmental timeline of ccRCC [13, 14, 21, 25]. Despite the disadvantages of these cellular and mouse models for ccRCC, their combined use has played a pivotal role in our current understanding of ccRCC disease processes and therapies. This highlights the importance of the choice of an experimental model and the need for additional representative models of ccRCC to investigate the mechanisms involved in disease progression and metastasis, as well as therapeutic resistance and testing [26]. These include cell lines in *in cellulo* or *in vitro* cell culture models, as well as *in vivo* models involving the use of CDX models, PDX models, and GEMMs to build upon our knowledge of ccRCC pathogenesis, in order to address the low survival rates seen in metastatic ccRCC. Furthermore, the choice of a model may also have a role in influencing the transcriptomic and signaling pathways and requires further characterization within an experimental context.

Here, we use a single RCC243 cell line harboring a *VHL* mutation that was established by selecting for a carbonic anhydrase IX (CA9) marker of ccRCC from the primary tumor of a ccRCC patient who was diagnosed with metastatic disease [10, 27]. We asked if this cell line could be used in various models of ccRCC, including metastasis. To investigate this, we generated the *in vitro* cell culture model of RCC243, *in vivo* orthotopic PDX tumor model with the implantation of the RCC243 PDX tumor into the renal capsule, *in vivo* orthotopic CDX tumor models using injections of RCC243 and RCC243‐tdTomato cell lines into the renal capsule, and *in vivo* metastatic tumor models using tail vein and intracardiac injections of the RCC243‐tdTomato cell line [23]. These RCC243 tumor models—cell culture, orthotopic tumors, and metastatic tumors—were then analyzed by histological and single‐cell RNA‐sequencing analyses. Furthermore, a comparison was made between the RCC243 models and ccRCC patient tumor datasets to determine their relevance to the patient outcome. Notably, the models generated using a single RCC243 cell line allowed for an unambiguous comparison between the models, providing intriguing insights into the impact of cellular context, platforms for greater understanding of the disease process, and a useful resource for future investigations.

## Materials and methods

### Cell culture

The RCC243 cells were previously generated from a ccRCC patient's primary tumor and were cultured as previously described [10, 23]. Briefly, cell culture plates were coated with collagen (Corning, 354236) prior to cell culture in IMDM, 10% FBS, and 5% penicillin–streptomycin. The cells were grown within an incubator set to 37 °C with 2% O_2_ and 5% CO_2_. The HEK293T cells were obtained from the American Type Culture Collection (RRID: CVCL_0063) and were cultured in DMEM, 10% FBS, and 5% penicillin–streptomycin in an incubator at 37 °C and 5% CO_2_.

### Transfection and lentiviral transduction

HEK293T cells were used for the transfection and lentiviral transduction process to integrate the pCDH‐EF1‐Luc2‐P2A‐tdTomato (pCDH‐EF1‐Luc2‐P2A‐tdTomato was a gift from Kazuhiro Oka; Addgene plasmid # 72486; http://n2t.net/addgene:72486; RRID: Addgene_72486) plasmid into the RCC243 cell line with the packaging plasmids, psPAX2 (psPAX2 was a gift from Didier Trono; Addgene plasmid # 12260; http://n2t.net/addgene:12260; RRID:Addgene_12260) and pMD2.G (pMD2.G was a gift from Didier Trono; Addgene plasmid # 12259; http://n2t.net/addgene:12259; RRID:Addgene_12259), in order to generate the RCC243‐tdTomato cell line to track the development of tumors within the mouse model. RCC243 cells were cultured concurrently, and the RCC243 media was changed for the 48‐h post‐transfection media of the HEK293T cells. The RCC243 cells were then cultured for another 24 h before using fluorescence‐activated cell sorting to confirm the integration of the plasmid.

### Fluorescence activated cell sorting

RCC243 cells positive for the tdTomato signal were sorted using the BD FACSAria IIu as previously described [23]. Briefly, the cells were sorted with the 100 μm nozzle with a sorting rate of approximately 1000 events per second.

### Generation of RCC243 mouse models

Non‐obese diabetic severe combined immunodeficiency IL‐2 receptor gamma knockout (NOD/SCID‐γ) mice (RRID: BCBC_4142) were used to generate the RCC243 mouse models based on the Canadian Council on Animal Care guidelines (protocol #1542). Ethical approval was acquired from the University Health Network Animal Care Committee (Animal Use Protocol #2178) [23]. The protocol for animal care included having an animal technician check the mice daily where if any of the mice appeared sick, this was reported to a clinical veterinarian to examine the animal. Recommendations would then be provided and implemented, including treatment for symptoms or euthanasia.

The *in vivo* orthotopic and metastatic models were generated using NOD/SCID‐γ mice, where RCC243 and RCC243‐tdTomato cell lines were used to create the orthotopic model through renal capsule injections. The RCC243 patient‐derived xenograft orthotopic model was created by implanting the tumor in the renal capsule, and the metastatic models were generated using tail vein and intracardiac injection methods of the RCC243‐tdTomato cell line to help study metastasis [28]. The renal capsule injections were performed by injecting a 1 : 1 PBS to Matrigel (Corning, 354234) mixture that contained one million RCC243 or RCC243‐tdTomato cells. The metastatic assay injections were performed by injecting 500 000 RCC243‐tdTomato cells in PBS for tail vein injections or 100 000 RCC243‐tdTomato cells in PBS for intracardiac injections. Afterwards, the orthotopic tumors were excised when the mice appeared pale and thin, and the metastatic tumors were excised when the bioluminescence signal of the tumors became saturated with *in vivo* bioluminescence imaging or when the mice appeared pale and thin. The mice were euthanized by cervical dislocation. The *in vivo* bioluminescence imaging was performed using the PerkinElmer Xenogen IVIS Spectrum Imaging System at the STTARR facility (University Health Network). The mice were injected with D‐luciferin (Perkin Elmer) and imaged 8 to 12 hours post‐injection with the Xenogen IVIS Spectrum Imaging System. Each cage of mice (containing at most 5 mice) was imaged at once every week or two weeks using an anesthetic procedure of 1–3% isoflurane with 1 L oxygen as an inhalant.

Additional tumors or portions of the tumors during the tumor dissociation process were fixed in formalin and then embedded in paraffin as formalin‐fixed paraffin‐embedded blocks. Tumor tissue sections were then prepared onto slides at the DDP Histo‐Biomarker Laboratory in the Princess Margaret Hospital, where H&E and Ki‐67 stains were prepared for each tumor model. The slides were then imaged using the Aperio AT2 brightfield scanner at the Advanced Optical Microscopy Facility (University Health Network).

### Tumor dissociation and preparation for single‐cell RNA‐sequencing

The tumors excised from the RCC243 mouse models were kept on ice and suspended in PBS before the dissociation process. The tumors were added to petri dishes with a dissociation mixture of IMDM, collagenase (VitaCyte, 001‐2030), protease (Sigma‐Aldrich, P5380‐100MG), and DNase I (Worthington, LS002006) [29, 30]. Mechanical and enzymatic dissociation of the tumor was performed at the same time using a scalpel and the dissociation mixture by alternating between incubation at 5% CO_2_ and 37 °C and pipetting in a biosafety cabinet for less than one hour in total. The cell suspension was then filtered through a 70 μm cell strainer, and 10% FBS in PBS kept at 4 °C was used to stop the enzymatic dissociation process. The suspension was centrifuged to pellet the cells, and ACK lysing buffer (Invitrogen, A10492‐01) kept at 4 °C was used to lyse red blood cells. The cells were then washed and suspended in 2% FBS in HBSS (FACS buffer) kept at 4 °C to check cell viability using trypan blue (Gibco, 15250‐061).

The cells were then prepared for magnetic‐activated cell sorting to remove the mouse cells. The cell suspension was blocked using mouse (RRID: AB_1163672) and rat (RRID: AB_1163629) IgG, with this unstained aliquot taken for subsequent flow cytometry confirmation of the depletion of mouse cells. Then, the suspension was stained with a 1 : 1000 dilution of mouse H2K[d]‐biotin antibody (RRID: AB_394922), a 1 : 200 dilution of mouse CD45‐biotin antibody (RRID: AB_394607), and a 1 : 200 dilution of mouse CD31‐biotin antibody (RRID: AB_397096) on ice. The cells were then washed, and anti‐biotin microbeads (Miltenyi Biotec, 130‐090‐485) were added to the sample on ice. Afterwards, the cells were washed again and labeled with a 1 : 400 dilution of Avidin‐PE‐Cyanine7 (Invitrogen, SA1012) on ice. Then, the cells were washed and filtered using a 70 μm cell strainer, with an aliquot acquired as the stained sample.

The cells were ready for magnetic‐activated cell sorting (Miltenyi Biotec, 130‐091‐051) afterwards, and 1 mL of cell suspension was added to one LS column for the depletion of mouse cells. The column was then washed with FACS buffer, and the eluant was collected representing the unlabeled human cells. Afterwards, the column was removed from the magnet, and the column was washed with FACS buffer to acquire the eluant representing the labeled mouse cells. An aliquot was taken from both samples for subsequent flow cytometry. The sample containing the human cells was suspended in IMDM and 10% FBS with a concentration of 1200 cells per μL for the single‐cell RNA‐sequencing submission process. The viability of the cells was checked prior to submission using trypan blue to ensure a cell viability greater than 80%.

### Cell fixation and confirmation of magnetic‐activated cell sorting by flow cytometry

The aliquoted samples were then fixed with Cytofix (BD, BD554655). Afterwards, the samples were stored within a box at 4 °C for subsequent flow cytometry to confirm the depletion of mouse cells from the sample submitted for single‐cell RNA sequencing. The aliquots were analyzed with the BD LSR Fortessa, and the aliquots were checked for PE‐Cyanine7 signal, with the tdTomato signal also being analyzed for RCC243‐tdTomato cells.

### Single‐cell RNA‐sequencing

The mouse cell depleted samples were processed at the Princess Margaret Genomics Centre with the 10X Genomics Chromium Single Cell 3′ Reagent Kit (v3.1) using the user guide. Briefly, the viability and cell count were checked with a hemocytometer, and then the GEMs were generated using the sample with the Chromium Next GEM Chip G. The sample cDNA was then generated using a thermal cycler and then purified and amplified to construct the cDNA library. Afterwards, the sample was sequenced using the Micro Flow Cell and the Illumina MiSeq platform to acquire the estimated cell count and targeted number of reads for subsequent sequencing with the S2 Flow Cell and Illumina NovaSeq 6000 platform.

### Data analysis

The *in vitro* cell culture and *in vivo* orthotopic and metastatic RCC243 single‐cell RNA‐sequencing datasets were processed using cell ranger 6.0 from 10× Genomics and the GRCh38‐2020‐A transcriptome to generate the filtered feature‐barcode matrix used for the downstream analysis [23]. The number of cells returned after sequencing for each RCC243 model based on the web summary output from the Cell Ranger pipeline included approximately 4221 cells for the RCC243 *in vitro* model, 4226 cells for the RCC243 *in vivo* orthotopic CDX model, 3912 cells for the RCC243‐tdTomato *in vivo* orthotopic CDX model, 4435 cells for the RCC243 PDX model, 3117 cells for the RCC243‐tdTomato *in vivo* metastatic tail vein model, and 2710 cells for the RCC243‐tdTomato *in vivo* metastatic intracardiac model. One tumor of each *in vivo* model when the tumors were ready to be excised and one cell culture dish for the *in vitro* model were sent for single‐cell RNA‐sequencing [23]. The single‐cell RNA‐sequencing datasets from Young *et al*. and Su *et al*. were also processed using cell ranger 6.0 and the GRCh38‐2020‐A transcriptome to ensure consistency for the analysis between the RCC243 tumor models and the patient datasets.

The seurat v4.4.0 package (RRID: SCR_001905), fgsea package (RRID: SCR_020938), clustree package (RRID: SCR_016293), mast package (RRID: SCR_016340), dplyr package (RRID: SCR_016708), vioplot package (RRID: SCR_019293), msigdbr package (RRID: SCR_022870), enhancedvolcano package (RRID: SCR_018931), ggplot2 package (RRID: SCR_014601), r.utils package, jsonlite package, scbp package (https://github.com/rnabioco/scbp), and patchwork package (RRID: SCR_024826) were used to conduct the single‐cell RNA‐sequencing analysis in R (RRID: SCR_001905) [31, 32, 33, 34, 35, 36, 37, 38, 39, 40, 41, 42, 43, 44, 45]. The code (provided within the Supplementary Data) used to conduct the analysis was based on the resources developed by the Harvard Chan Bioinformatics Core, Babraham Bioinformatics, and Seurat vignettes. The quality control for the dataset was originally conducted by analyzing each dataset individually to determine the minimum and maximum number of genes (nFeature_RNA). The inflection point for the minimum and the maximum were chosen as the cutoffs. Within the analyses with the integration of the datasets, the cells were filtered by choosing the lowest minimum number of genes cutoff and the highest maximum number of genes cutoff. The cells were also filtered by setting a mitochondrial gene percentage cutoff of 40%. Afterwards, 41 principal components were used for the analysis using an approach to determine which principal components cumulatively contribute more than 90% of the standard deviation and where the individual principal component contributes less than 5% of the standard deviation [46]. For the RCC243 tumor model analysis, cells were additionally filtered by removing mouse cells and cells with library issues based on the quality control metrics. For the RCC243 tumor model and patient ccRCC dataset analysis, cells were filtered by removing immune, endothelial, and fibroblast cells within the patient datasets, as well as filtered by removing mouse cells and cells with library issues within the RCC243 datasets. Batch correction was performed in this analysis using Harmony (RRID: SCR_022206) with ‘max_iter = 1’ following the filtering of the cells [47]. Differential gene expression analysis was performed using Seurat and MAST to generate the list of differentially expressed genes between the clusters [31, 34]. The gene list generated using MAST was then used to conduct gene set enrichment analysis using fgsea for the RCC243 models [45].

For the spatial transcriptomics analysis of the Meylan *et al*. dataset, the analysis was performed based on the Seurat vignette [48]. Further analysis to determine correlations and differential expression between the areas within the spatial dataset was performed using the Loupe Browser 8.1.2 with the conversion of the Seurat file using LoupeR.

The Kaplan–Meier survival analysis was conducted using the cBio Cancer Genomics Portal and the TCGA KIRC dataset [49, 50, 51, 52]. The top 15 differentially expressed genes from the orthotopic and metastatic datasets were used to create the groups for the analysis. Overexpression of the genes that were available in the TCGA KIRC samples with mRNA data (with “EXP>=2” were used for both the orthotopic and metastatic datasets to create the altered groups that were compared to the unaltered groups, which did not have overexpression of these genes). The altered groups contained samples that had at least one of the genes overexpressed within the analysis.

## Results

### RCC243 recapitulates a clear cell phenotype in *in vivo* orthotopic and metastatic tumor models of ccRCC

**Fig. 1:**
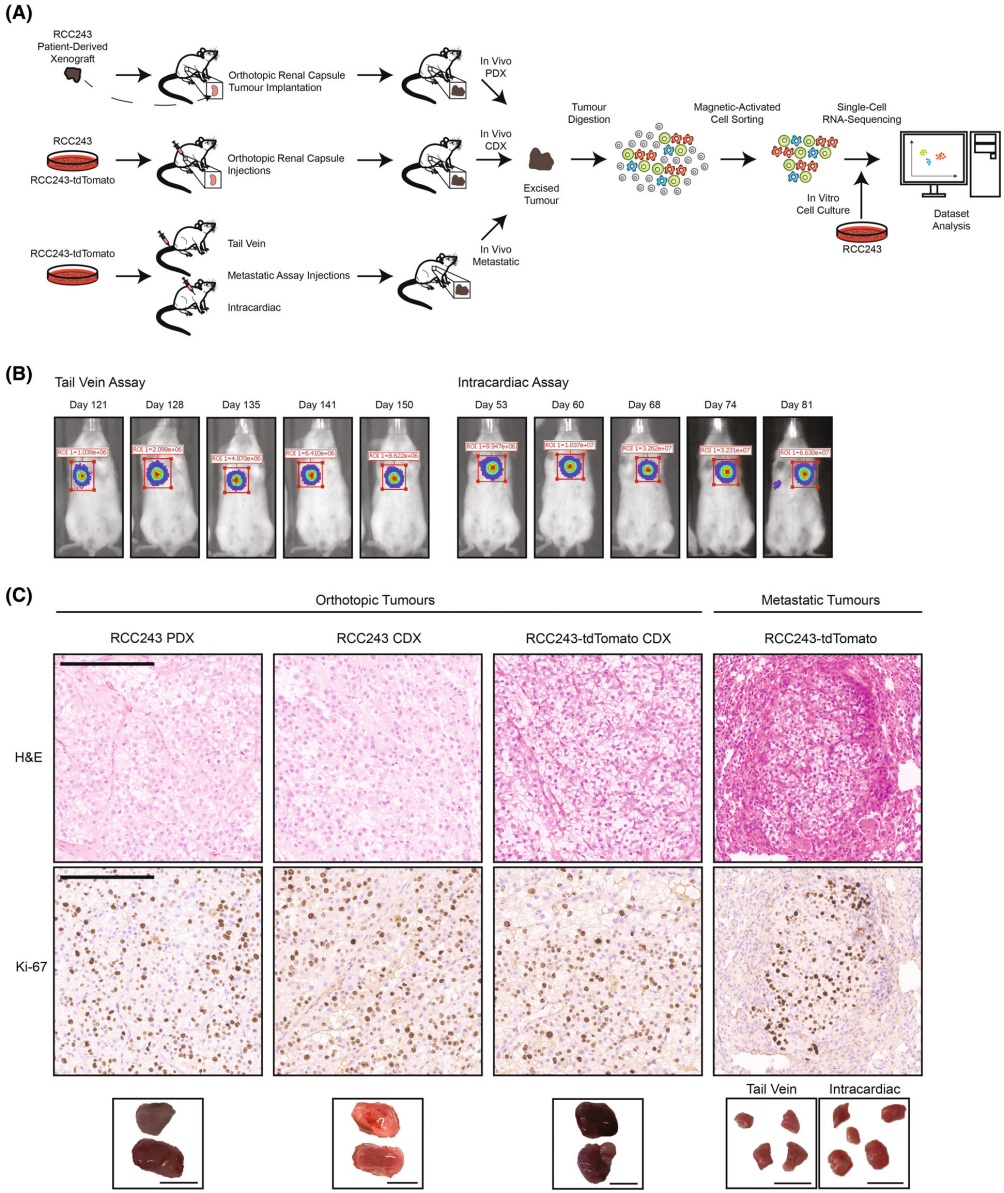
*In vivo* orthotopic and metastatic model of ccRCC using RCC243 that recapitulates a clear cell phenotype. (A) Flow chart of the experimental process to generate the RCC243 tumor models and single‐cell RNA‐sequencing datasets. The RCC243 PDX was implanted into the renal capsule to generate an *in vivo* PDX orthotopic tumor. The RCC243 or RCC243‐tdTomato cell lines were injected into the renal capsule to generate the *in vivo* CDX orthotopic tumors. The RCC243‐tdTomato cell line was injected into the tail vein or intracardially through metastatic assay injections to generate the *in vivo* metastatic tumors. The RCC243 cell line was also grown *in vitro* to generate the *in vitro* cell culture model [23]. The tumors were dissociated and depleted of mouse cells prior to sequencing. (B) Representative *in vivo* bioluminescence images of the tumors within the lungs of NOD/SCID‐γ mice following tail vein (*n* = 15) or intracardiac metastatic assay (*n* = 15) injections of the RCC243‐tdTomato cell line. (C) Representative images of H&E‐stained and Ki‐67‐stained tumor sections (*n* = 1 each for RCC243 PDX, RCC243 CDX, RCC243‐tdTomato CDX, and RCC243‐tdTomato Metastatic) (scale bars represent 200 μm) and images of the excised orthotopic and metastatic tumors (*n* = 2 for each orthotopic tumor model and *n* = 3 for each metastatic tumor model) (scale bars represent 1 cm). The orthotopic tumors represent the *in vivo* RCC243 PDX model, *in vivo* RCC243 CDX model, and the *in vivo* RCC243‐tdTomato CDX model. The metastatic tumors represent the *in vivo* RCC243‐tdTomato metastatic model that is generated through tail vein and intracardiac injections.

In order to investigate the RCC243 cell line and to characterize its ability to form metastatic tumors, a variety of models for single‐cell RNA sequencing were generated to investigate possible differences that may exist between *in vitro* and *in vivo* settings (Fig. 1A). This included the *in vitro* cell culture model as well as *in vivo* models using NOD/SCID‐γ mice through orthotopic renal capsule injections with RCC243 and the orthotopic renal capsule implantation with the corresponding PDX model (Fig. 1A) [23]. There was an initial attempt to determine whether metastatic tumors would form following orthotopic renal capsule injections as the original patient developed metastatic disease [10]. However, it was difficult to observe the development of metastatic tumors, and this did not occur prior to the mice becoming visibly sick from the orthotopic tumor. Thus, the RCC243‐tdTomato cell line was developed to allow us to track tumor development (Fig. S1 and Fig. 1A). This was generated with RCC243 through the integration of a plasmid expressing luciferase and the tdTomato fluorescent protein. This cell line was then used for orthotopic renal capsule injections as a comparison with the RCC243 cell line, and RCC243‐tdTomato was used for metastatic assay injections including tail vein and intracardiac injections to visualize the development of metastatic tumors (Fig. 1A) through the use of *in vivo* bioluminescence imaging (Fig. 1B) [28]. Metastatic tumors within the lungs of mice formed following both types of metastatic assay injections, with tumors forming faster following intracardiac injections (Fig. 1B and Fig. S2).

As tumors developed in all of the *in vivo* models, we asked whether there were differences in the tumor histology between each experimental setting. After excision of the tumors from the mouse models, a portion of each tumor was prepared for H&E staining, which revealed that the clear cell phenotype was maintained in both the orthotopic and metastatic tumors (Fig. 1C). Furthermore, Ki‐67 staining was performed to visualize the proliferative activity of tumors, and it revealed similar staining between all tumor models (Fig. 1C).

### Single‐cell RNA‐sequencing highlights the role of the experimental model on differential gene expression

After generating the *in vivo* orthotopic and metastatic models of RCC243, the tumors from the mice were excised, and the samples were dissociated into single‐cell suspensions (Fig. 1A). As the tumors also included mouse cells, magnetic‐activated cell sorting was performed to remove the mouse cell fraction prior to the submission of the sample for single‐cell RNA sequencing. The efficiency of the dissociation and depletion process was confirmed using flow cytometry following each tumor dissociation to ensure that most of the submitted sample contained human cells from the RCC243 model (Fig. S1). The *in vitro* model single‐cell RNA sequencing data of RCC243 was previously generated from single‐cell suspensions of RCC243 cells grown in cell culture [23]. The reads were processed using cell ranger 6.0, and the quality control and downstream analyses were performed using the Seurat package [31] (Fig. S3). The generated data allowed for an investigation of the expression of genes within individual cells in both *in vitro* cell culture and *in vivo* orthotopic and metastatic tumors, which enabled a comparison to be made between the gene expression profiles of the various models of RCC243.

**Fig. 2:**
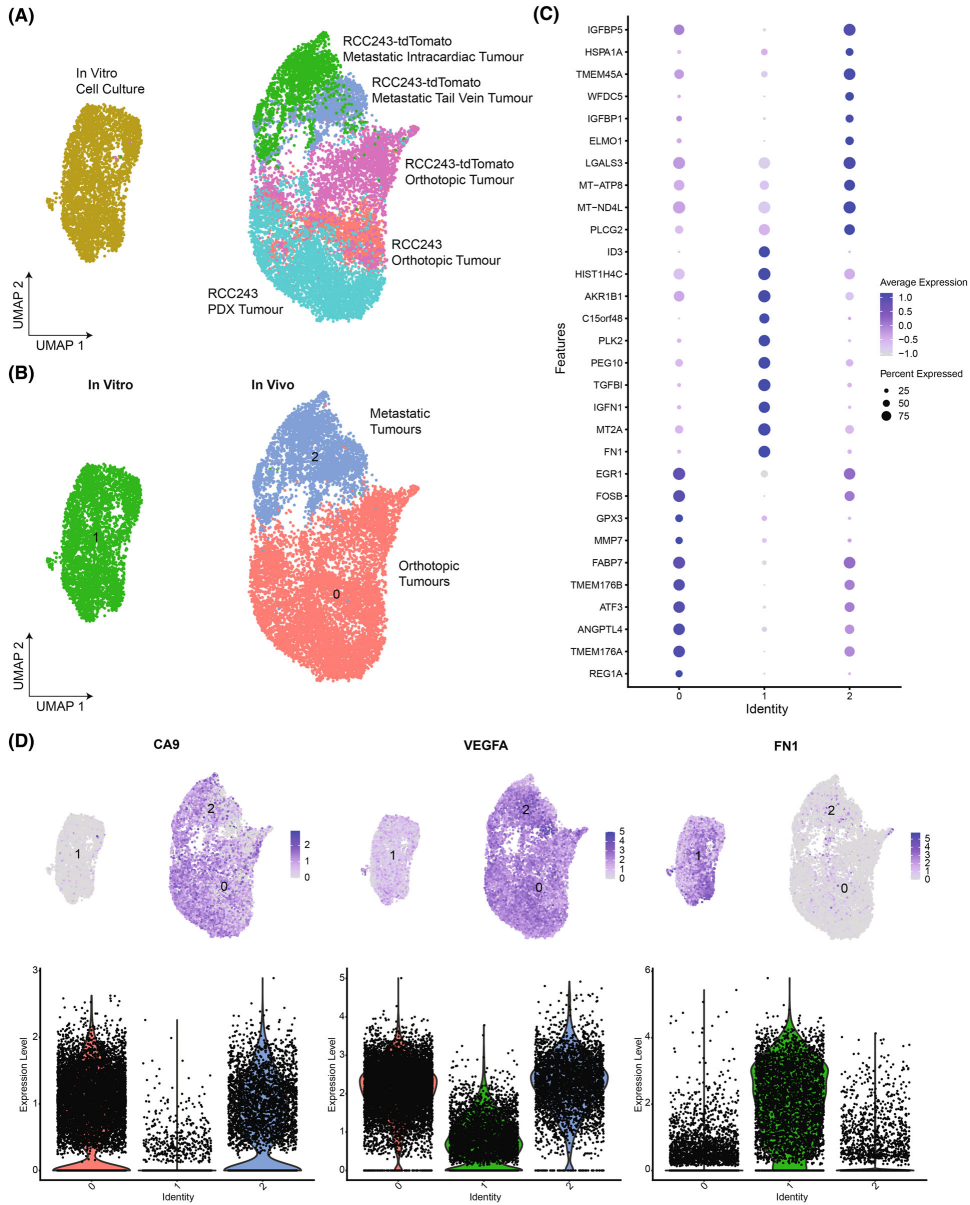
Single‐cell RNA sequencing highlights the role of the experimental model on differential gene expression. The datasets were combined and analyzed using the Seurat package. (A) The UMAP of the RCC243 tumor models generated based on the flow chart in Fig. 1A with groups showing the origin of the cells [23]. (B) The UMAP after clustering of the models reveals that cells mainly cluster based on their localization *in vitro* and *in vivo*. Cluster 0 represents the orthotopic tumor models, cluster 1 represents the *in vitro* cell culture model, and cluster 2 represents the metastatic tumor models. (C) A dot plot of the top 10 most differentially expressed genes within each cluster of cells. (D) A selection of prognostic markers from the Protein Atlas (*CA9*, *VEGFA*, and *FN1*) with cellular expression plotted on the UMAPs and as violin plots based on cluster identity.

As a result, we asked whether there were differences in the transcriptional profiles of cells based on the model type. The cells were found to be grouped together according to whether they were from an *in vitro* or *in vivo* model (Fig. 2A), and clustering of the dataset using resolution 0.1 revealed three clusters based on whether cells were grown in *in vitro* cell culture, within the orthotopic renal capsule, or within the lungs following metastatic tail vein or intracardiac assay injections (Fig. 2B). In addition, we also asked whether there were genes that were differentially expressed based on the three clusters that were formed, and a generated list of the topmost differentially regulated genes in each cluster revealed that the greatest gene expression differences existed between the *in vitro* and *in vivo* clusters (Fig. 2C and Fig. S4), which highlights the role of the experimental model on the transcriptional expression of cells. Furthermore, we also investigated a selection of prognostic markers in ccRCC including *CA9*, *VEGFA*, and *FN1*, which highlighted a model‐specific expression pattern with an upregulation of *CA9* and *VEGFA* within the *in vivo* models and an upregulation of *FN1* within the *in vitro* model (Fig. 2D).

### Gene set enrichment analysis reveals the differential expression of pathways between RCC243 models

**Fig. 3:**
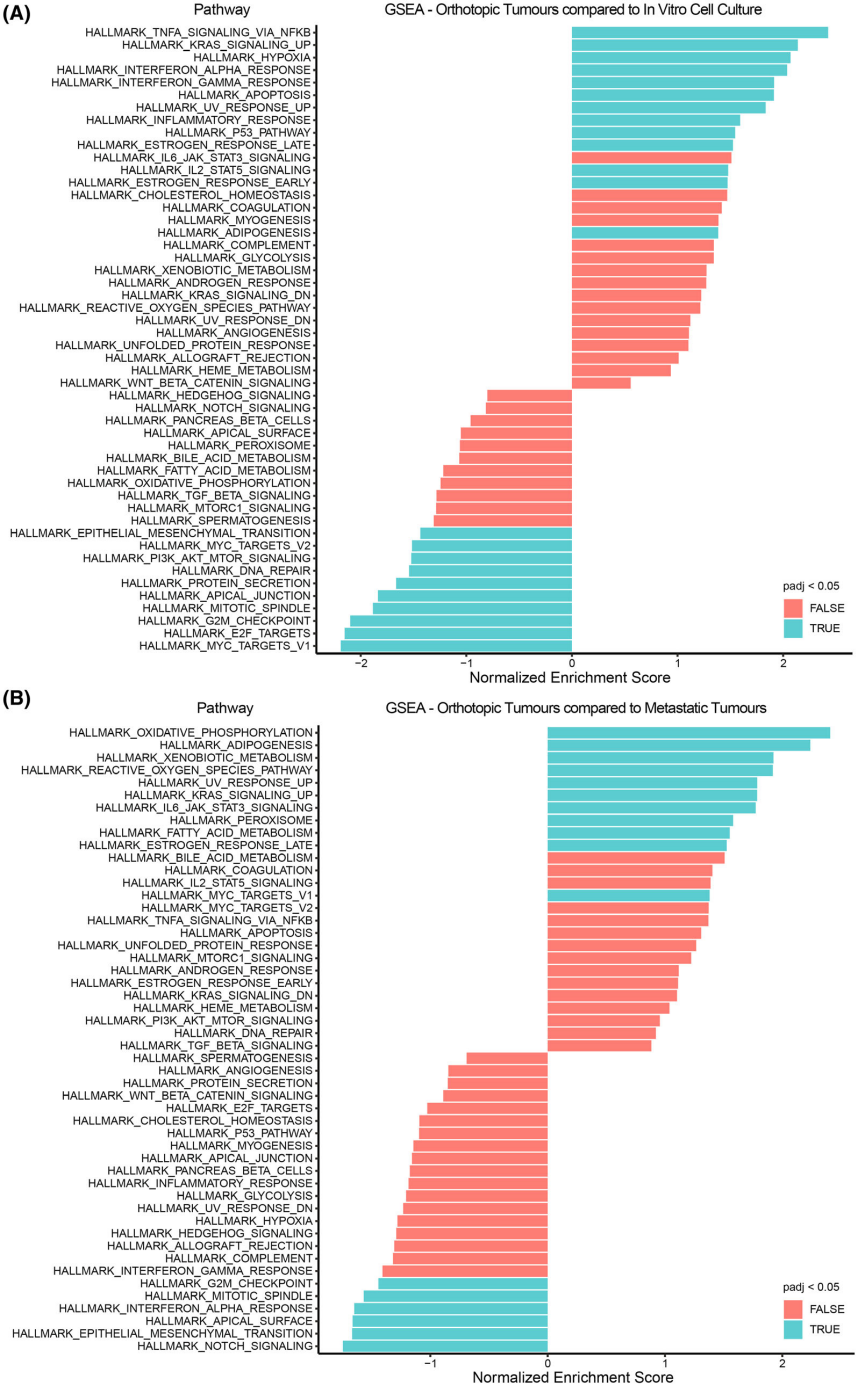
Gene set enrichment analysis between the RCC243 tumor models reveals differential gene signatures. The hallmark pathways were from the Molecular Signatures Database. The most enriched pathways for each cluster in this comparison are shown based on the normalized enrichment score. The GSEA was conducted with a list of genes generated using MAST based on genes that were upregulated in the orthotopic tumor dataset compared to the *in vitro* cell culture dataset or genes that were upregulated in the orthotopic tumor dataset compared to the metastatic tumor dataset. (A) *In vivo* orthotopic tumor cluster comparison with the *in vitro* cell culture cluster (with the most enriched pathways in the orthotopic cluster shown at the top and the enriched pathways in the *in vitro* cluster shown at the bottom). (B) *In vivo* orthotopic tumor cluster comparison with the *in vivo* metastatic tumor cluster (with the most enriched pathways in the orthotopic cluster shown at the top and the enriched pathways in the metastatic cluster shown at the bottom). GSEA was performed using the fgsea package (Figs S5 and S6), which involves the use of Benjamini‐Hochberg correction to acquire the adjusted *P*‐values, and pathways were deemed significant based on an adjusted *P*‐value < 0.05.

As there were differences in gene expression between the models of RCC243, we asked whether these differentially expressed genes were involved together in cellular pathways. To answer this question, a list of differentially expressed genes was generated between the *in vivo* orthotopic and *in vitro* cell culture datasets, as well as between the *in vivo* orthotopic and *in vivo* metastatic tail vein and intracardiac datasets using MAST [34] (Fig. 3A,B and Tables S1 and S2). Gene set enrichment analysis (GSEA) was then performed using the fgsea package and the hallmark gene sets from the Molecular Signatures Database [45, 53, 54] (Figs S5 and S6).

The GSEA results revealed that there was a significant enrichment of pathways that included TNF‐α signaling through NFκB, KRAS signaling, hypoxia, IFN‐α and IFN‐γ responses, apoptosis, UV radiation response, inflammatory responses, p53 responses, estrogen responses, signaling through IL‐2/STAT5, and adipogenesis within the orthotopic tumor dataset in comparison to the *in vitro* cell culture dataset (Fig. 3A and Fig. S5). In contrast, the pathways that were significantly enriched within the *in vitro* cell culture dataset in this comparison included MYC regulated genes, E2F regulated genes involved in the cell cycle, G2M checkpoint genes, mitotic spindle assembly genes, apical junction complex genes, secretory protein genes, DNA repair genes, genes involved in PI3K/AKT/mTOR signaling, and the epithelial‐mesenchymal transition (Fig. 3A and Fig. S5). Furthermore, the GSEA results also revealed differentially enriched pathways between the orthotopic and metastatic datasets, which showed an enrichment of pathways involved in oxidative phosphorylation, adipogenesis, drug metabolism, the response to reactive oxygen species, UV radiation response, KRAS signaling, IL‐6/JAK/STAT3 signaling, peroxisomes, fatty acid metabolism, late estrogen response, and MYC responses for the orthotopic dataset (Fig. 3B and Fig. S6). Whereas the pathways that were enriched within the metastatic tumor dataset included notch signaling, the epithelial‐mesenchymal transition, apical surface proteins, IFN‐α response, mitotic spindle assembly, and G2M checkpoint genes (Fig. 3B and Fig. S6). Overall, there were significantly more differentially enriched pathways within the GSEA between the *in vivo* orthotopic tumor and *in vitro* cell culture models when compared with the GSEA between the *in vivo* orthotopic tumor and *in vivo* metastatic tumor models (Fig. 3A,B), which is consistent with the greater amount of differential gene expression observed between the *in vivo* orthotopic tumor and *in vitro* cell culture datasets.

### Analysis of RCC243 tumor model datasets with patient tumor datasets reveals the conservation of some prognostic markers of ccRCC

**Fig. 4:**
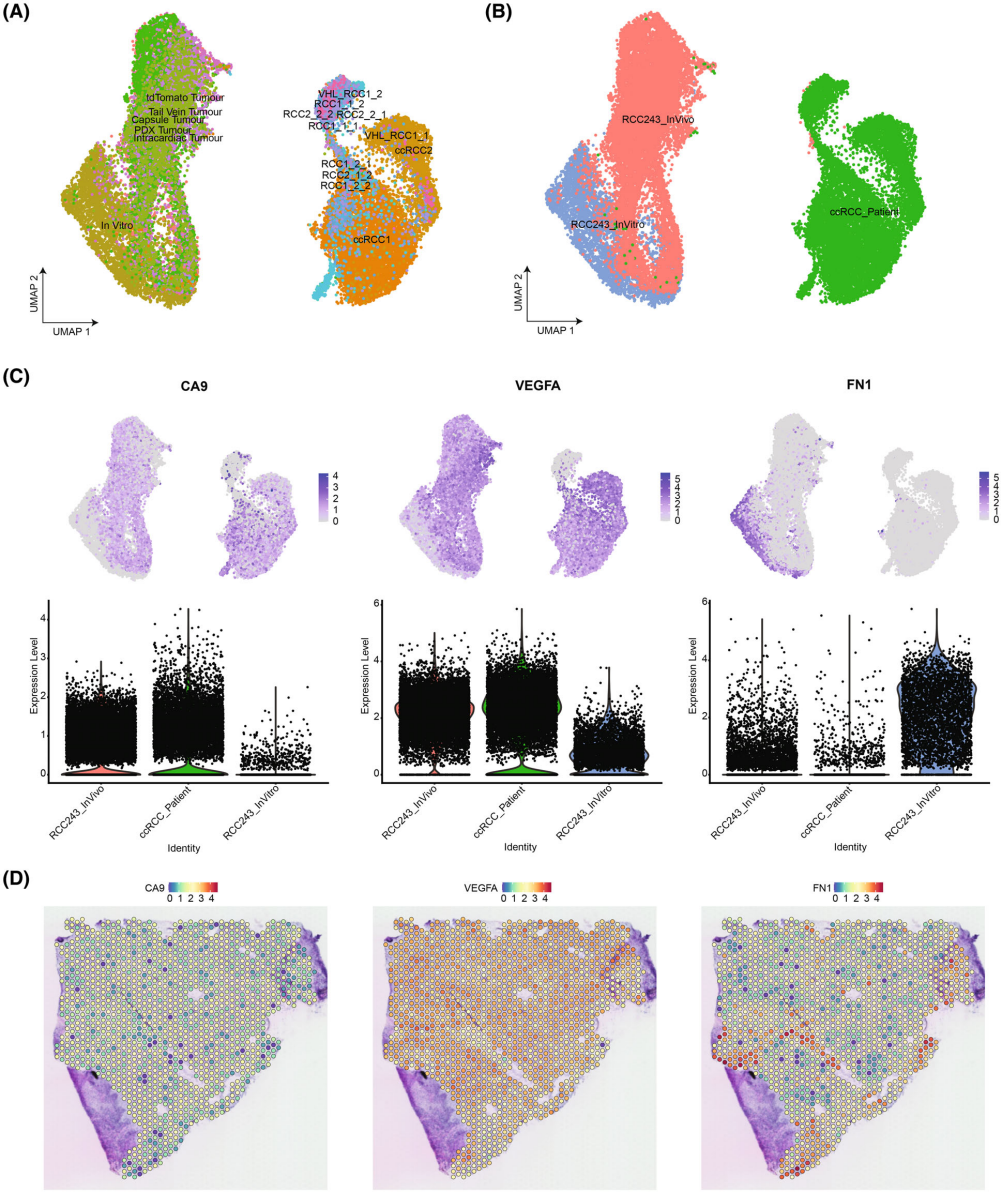
The integration of RCC243 tumor models and patient ccRCC tumor datasets reveals some conservation of prognostic markers. The datasets were combined and analyzed using Seurat. Batch correction was performed using Harmony, with the non‐batch‐corrected UMAP shown in Fig. S7. (A) The UMAP of the RCC243 tumor models and patient ccRCC tumor datasets from Young *et al*. (RCC1_1_1, RCC1_1_2, RCC1_2_1, RCC1_2_2, RCC2_1_2, RCC2_2_1, RCC2_2_2, VHL_RCC1_1, and VHL_RCC1_2) and Su *et al*. (ccRCC1 and ccRCC2) with groups based on cellular origin [23, 55, 56]. (B) Clustering of the datasets following batch correction with Harmony shows that RCC243 tumor models cluster together and patient ccRCC tumor datasets cluster together separately. (C) A selection of prognostic markers from the Protein Atlas (*CA9*, *VEGFA*, and *FN1*) highlights that *CA9* and *VEGFA* are upregulated in the patient tumors and RCC243 *in vivo* tumor datasets, whereas *FN1* is upregulated in the RCC243 *in vitro* cell culture dataset. Cellular expression levels are presented within the UMAP and as violin plots based on cluster identity. (D) Spatial transcriptomics dataset of a ccRCC tumor analyzed using Seurat, with the relative expression of *CA9*, *VEGFA*, and *FN1* from Meylan *et al*. The expression levels are shown using a heatmap, with dots representing locations in the tumor section [48].

Following our investigation of the RCC243 datasets, we asked whether this model was representative of the patient tumor setting. We examined whether there were conserved markers that were present within the RCC243 tumor models (cell culture, orthotopic tumors, and metastatic tumors) and ccRCC patient tumors. The RCC243 datasets were integrated with patient datasets from Young *et al*. and Su *et al*. in order to determine similarities and differences in gene expression [55, 56]. However, as the patient tumor datasets contain a wide variety of cell types including immune, endothelial, and fibroblast cells, these cells were first removed from our analysis based on the expression of *PTPRC* (*CD45*), *ESM1*, and *TAGLN* respectively [57, 58]. The subsequent analysis revealed that the cells mainly clustered based on the tumor sample and dataset (Fig. S7), which was expected due to the different sequencing technologies employed as well as the heterogeneity of ccRCC tumors. After batch correction was performed using Harmony, this revealed that the patient tumor datasets clustered similarly together (Fig. 4A,B, Figs S7 and S8).

We looked at the expression of three prognostic markers: *CA9*, *VEGFA*, and *FN1* that were previously used when characterizing the RCC243 models (Fig. 2D). A similar pattern of gene expression persisted, where *CA9* and *VEGFA* were upregulated within the *in vivo* RCC243 tumor models and ccRCC patient datasets, whereas *FN1* was upregulated within cells grown in the *in vitro* RCC243 cell culture model (Fig. 4C). A similar pattern of expression for *CA9* and *VEGFA* was also identified in a spatial transcriptomics dataset of a ccRCC tumor from Meylan *et al*. [48] (Fig. 4D). However, there also appeared to be a higher relative expression of *FN1*, as well as an increased *FN1* expression in areas with low *CA9* expression (Fig. S9), which could potentially be a result of the heterogeneous cell population within the tumor section, whereas the single‐cell datasets only contain the tumor cells. Further differential expression analysis was performed between the patient tumor datasets and the RCC243 tumor models, which revealed some overlap of gene expression among the most differentially expressed genes (Fig. S7). Overall, the results highlight that some prognostic markers of ccRCC are conserved only within the patient or *in vivo* RCC243 tumor setting, which suggests that the experimental model can influence the expression of prognostic markers that may have implications for therapeutic and mechanistic investigations.

### Top differentially expressed genes in the metastatic dataset are correlated with a worse prognosis in ccRCC patients

**Fig. 5:**
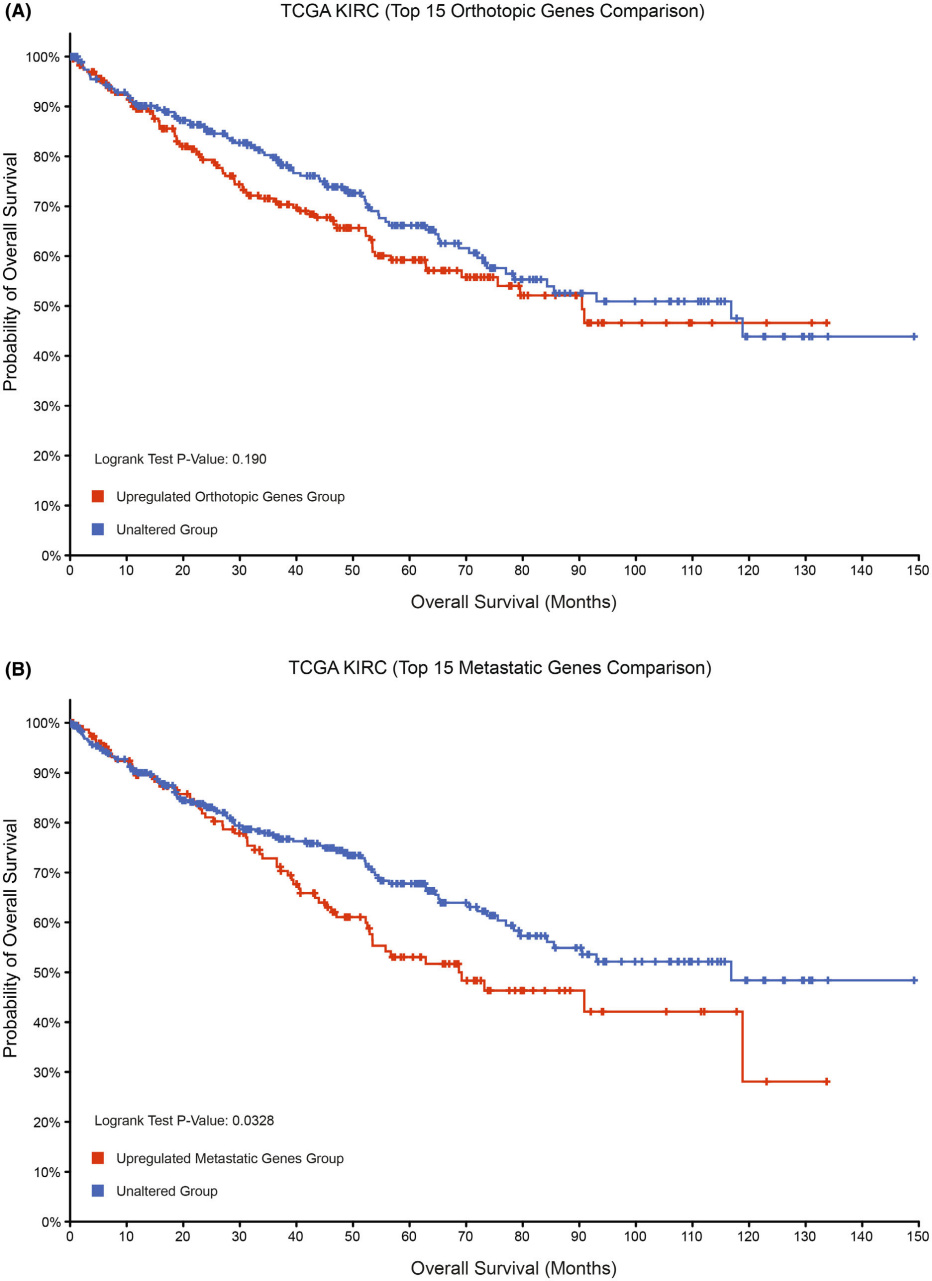
The top differentially expressed genes in the metastatic dataset are correlated with a worse prognosis in ccRCC patients based on the TCGA KIRC dataset. A comparison was conducted between the top differentially regulated genes of the RCC243 *in vivo* orthotopic and metastatic tumor datasets using the cBio Cancer Genomics Portal to look at the role of these genes in patient survival based on the TCGA KIRC dataset with available RNA‐sequencing data (*n* = 510). (A) Kaplan–Meier survival analysis performed based on the top 15 differentially expressed genes within the orthotopic tumor dataset. (B) Kaplan–Meier survival analysis performed based on the top 15 differentially expressed genes within the metastatic tumor dataset.

Following the identification of genes and pathways that were upregulated in the RCC243 tumor model datasets, we next asked whether the expression of these genes had implications for patient survival. To investigate this question, we used the top differentially expressed genes between the orthotopic and metastatic tumor datasets with the cBio Cancer Genomics Portal, as well as the TCGA KIRC dataset that contains data on ccRCC patients [49, 50, 51, 52]. This enabled us to perform a Kaplan–Meier survival analysis, which visualized the survival rate of patients with tumor samples that had an upregulated expression of the genes identified in our list of the top 15 differentially expressed genes within the *in vivo* orthotopic and metastatic datasets (Fig. S4). This was compared with the unaltered group, which consisted of patients with tumors that did not display an upregulation of one or more of these genes. The results revealed that there was no significant difference in survival between patients with tumors that had an upregulation of genes within the *in vivo* RCC243 orthotopic tumors and those that did not have an upregulation of these genes (Fig. 5A). In contrast, tumors that had an upregulated expression of genes within the *in vivo* RCC243 metastatic tumors were associated with a worse overall survival compared to tumors that did not have an upregulation of these genes (Fig. 5B). These results show that the most differentially expressed genes identified in our RCC243 metastatic tumor dataset correlate with a more aggressive disease phenotype.

## Discussion

In this study, we generated ccRCC tumor models using a single patient‐derived RCC243 cell line, which was able to form metastatic tumors following tail vein or intracardiac metastatic assay injections while maintaining its clear cell phenotype. This is important as more models are required to understand disease progression and metastatic development, as well as to model the heterogeneity of ccRCC tumors [13, 26].

In our single‐cell RNA‐sequencing analysis, we found that the cells from the RCC243 tumor models (cell culture, orthotopic tumors, and metastatic tumors) cluster based on the type of model, such as *in vitro* and *in vivo*, and there is further separation *in vivo* between orthotopic and metastatic tumors. This demonstrates that the choice of experimental model has an influence on the transcriptional profile. This is supported by the differential expression of prognostic markers in ccRCC based on the model type, such as *CA9*, *VEGFA*, and *FN1* [59, 60]. Furthermore, an analysis of the datasets using Shannon entropy to quantify the transcriptional heterogeneity of the clusters revealed that the orthotopic tumors had the largest entropy value, followed by the metastatic tumors and then the *in vitro* cell culture dataset (Fig. S10). This suggests that the cells adopt the most cell states in the orthotopic setting and that there may be a transcriptional profile involving the top differentially regulated genes that favors metastasis, as the metastatic tumor dataset exhibits less heterogeneity. In contrast, the cells grown in *in vitro* cell culture adopt the least amount of cell states and appear to exhibit a very homogeneous transcriptional program. Overall, this highlights the importance of the choice of a model because differences exist between *in vitro* cell culture and *in vivo* tumor models.

We also observed that pathways were differentially regulated between the tumor model types [53, 54]. Our comparisons showed that the most significant differences in pathway expression were between the *in vivo* orthotopic and *in vitro* cell culture tumor models. This included pathways involved in inflammatory responses, which may be involved in tumor development [61, 62]. In addition, there was an enrichment of hypoxic signaling in the orthotopic dataset despite the cell line having inactivating mutations for *VHL*, which may be a consequence of other pathways such as the unfolded protein response and KRAS signaling that can upregulate genes involved in the hypoxic response, such as *CA9* and *VEGFA*, independent of HIF [63, 64, 65]. There was also an enrichment in pathways associated with interferon signaling, which has been implicated in other cancers by supporting tumorigenesis [18, 66]. Furthermore, pathways such as IL‐2/STAT5 signaling were enriched, which have been implicated in inflammatory responses, as well as the activation of p53 and apoptotic pathways [67]. Other pathways that were enriched may also be involved in tumor progression including the estrogen response pathways, as well as the adipogenesis pathway, which may help contribute to the clear cell morphology seen in ccRCC [68, 69, 70].

These pathways differ from the *in vitro* cell culture dataset where there is an enrichment in pathways that are collectively associated with the cell cycle, including cell division and proliferation. This is consistent with the cells being grown in cell culture, where they can rapidly proliferate, whereas the tumor setting may have restrictions on space and nutrient access. Other pathways that were enriched included genes that encode secretory proteins in the *in vitro* cell culture dataset, which suggests a role in the formation of the extracellular matrix as well as in intercellular communication. This may show that the *in vitro* cell culture model adopts a more mesenchymal phenotype, as the epithelial‐mesenchymal transition (EMT) pathway was also enriched, which was previously found in tumors with an upregulation of *FN1* [57, 70].

We conducted the same analysis between the *in vivo* orthotopic and metastatic tumor datasets, which provided insight into the gene expression profile that is involved in the establishment and survival of metastatic tumors. Many of the pathways that were differentially expressed and upregulated in the orthotopic tumor dataset include those involved in metabolism, and this may suggest that the cells within the metastatic dataset are less metabolically active, which could be a result of how the metastatic tumors are still expanding due to their smaller size as well as the differences in nutrient availability between the environments of the lung and kidney [71]. The pathways enriched in the metastatic tumors include those involved in the cell cycle as well as the EMT, which is consistent with this model, as this pathway has been implicated in metastatic processes [72]. The enrichment of the cell cycle and EMT pathways was also observed in the comparison between the *in vitro* cell culture and orthotopic tumor datasets. As the metastatic tumors develop from the injection of cells grown in cell culture, this appears to highlight how some pathways remain conserved between the *in vitro* cell culture and metastatic tumor datasets. However, within our analysis, the differences that existed between the *in vivo* orthotopic and metastatic tumor models were not as significant as the comparison between the *in vivo* orthotopic and *in vitro* cell culture tumor models.

We also asked if the RCC243 cell line in our models is representative of patient tumors. We found that the expression profile of the prognostic marker genes: *CA9*, *VEGFA*, and *FN1*, observed in *in vivo* orthotopic and metastatic tumor datasets was similar to the patient tumor datasets, whereas *FN1* was only upregulated in the *in vitro* cell culture dataset. This also appeared to be consistent with the ccRCC patient tumor within the spatial transcriptomics dataset; however, there was a higher relative expression of *FN1*, which may reflect the diversity of cells within the tumor microenvironment [48]. Interestingly, it appeared that *FN1* is more highly upregulated in areas with lower *CA9* expression (Fig. S9) and areas with higher FN1 expression were also associated with an increased expression of fibroblast markers (Fig. S11), which may be a result of the presence of tumor cells that adopt a more mesenchymal phenotype as well as fibroblasts [57, 73]. The gene expression profile of the RCC243 tumor models and the patient tumors still display differences based on the clustering profile of the cells. This can be a result of the heterogeneity observed in ccRCC tumors, which may also involve differences in the cellular copy number profiles and the impact of the immune system in the patient setting [13]. We also conducted an analysis using patient tumor datasets and patient survival statistics based on the gene signatures that we observed for the *in vivo* orthotopic and metastatic tumor models. This revealed that the top differentially regulated genes within the metastatic tumor dataset were significantly associated with worse patient survival compared to the genes in the orthotopic tumor dataset that showed no significant difference when using the TCGA dataset [49, 52]. Within this list of genes in the metastatic tumor dataset, individual genes such as *TMEM45A*, *IGFBP1*, *IGFBP5*, and *MALAT1* appeared to be associated with a worse patient survival. This may suggest that the RCC243 cells that form metastatic tumors have a gene signature that is more aggressive or resistant, and this would be a model that can be used for further characterization of metastatic processes.

We recognize that there are limitations based on the model and methods used within this study. Although we have used a cell line model that remains similar based on its genetic profile to its original patient tumor [10], this still represents one model in ccRCC, which is characterized by significant levels of heterogeneity [3, 13]. The NOD/SCID‐γ mouse models that were used represent an immunocompromised model that would not recapitulate the immune phenotypes and interactions that are observed in ccRCC patient tumors [6]. However, despite this, this cell line model can still be used to investigate tumor progression and metastatic mechanisms, and it would represent an additional model that can be used in future investigations with currently established cell lines. Although we recognize that metastatic assay injections bypass the necessary process whereby primary tumor cells leave their environment, it is a useful approximation of metastasis as it still enables us to investigate genes and pathways involved in cell survival and colonization [28].

Other experimental limitations include the dissociation process for tumors that is required for single‐cell RNA‐sequencing. The use of enzymatic and mechanical dissociation can result in artifacts in transcriptional expression that is a consequence of cell stress during this process [74, 75]. The variation in protocols can also introduce batch effects when comparing samples [76]. However, our experimental setup uses one cell line in different model settings, and a comparison of gene expression between the original RNA‐sequencing data of the RCC243 primary tumor and the *in vitro* cell culture model reveals a similar pattern in gene expression with our single‐cell RNA‐sequencing data [10]. There are also limitations associated with single‐cell RNA‐sequencing as transcript expression does not always correlate with protein expression within a cell [77], which mediates the function of the gene. As a result of this, further experiments involving the use of western blots and additional molecular investigations would be required to validate and provide further support for the conclusions of this study.

Overall, our study characterizes the transcriptional profiles of a single primary tumor‐derived ccRCC cell line called RCC243 within different model settings, which can be used as a resource for future analyses. We showed that this cell line can form metastatic tumors displaying a prominent clear cell phenotype and that there were significant differences in the genes and pathways that were expressed between the various RCC243 tumor models (i.e., cell culture, orthotopic tumors, and metastatic tumors). This underscores the role of the experimental model type in influencing the cellular phenotype, which has implications for the choice of the experimental model used for mechanistic investigations and therapeutic testing. Furthermore, we identified that some prognostic markers were conserved between the RCC243 tumor models and patient tumors, and that the genes that were upregulated in the metastatic tumor datasets were associated with worse prognosis, which provides support for the use of this cell line and animal model in future investigations of ccRCC pathogenesis.

## Conflict of interest

The authors declare no conflict of interest.

## Peer review

The peer review history for this article is available at https://www.webofscience.com/api/gateway/wos/peer‐review/10.1002/2211‐5463.70022.

## Author contributions

Conceptualization, CMR and MO; Methodology, RH, LK, AG, JM, FGF, MSI, LA, SAY, CMR, and MO; Formal Analysis, RH, SAY, CMR, and MO; Investigation, RH, LK, AG, JM, MSI, LA, SAY, CMR, and MO; Resources, MSI, LA, SAY, CMR, and MO; Writing – Original Draft, RH and MO; Writing – Review and Editing, RH, LK, AG, JM, FF, MSI, LA, SAY, CMR, and MO; Visualization, RH, SAY, CMR, and MO; Supervision, MSI, LA, SAY, CMR, and MO; Funding Acquisition, MO.

## Supporting information


**Fig. S1.** Creation of the RCC243‐tdTomato cell line and confirmation of the depletion of mouse cells for single‐cell RNA‐sequencing. (A) A comparison of a healthy mouse kidney and a kidney affected by the injection of RCC243. (B) The confirmation of the generated RCC243‐tdTomato cell line by fluorescence activated cell sorting. (C) Confirmation of the depletion of mouse cells that were stained with the PE‐Cyanine7 fluorochrome following the tumour dissociation process for each model of RCC243. The analysis for the RCC243‐tdTomato intracardiac tumour sample may have been contaminated with cells present within the lineage positive fraction that contained the depleted mouse cells as it was analyzed immediately prior to the lineage negative fraction.
**Fig. S2.** Tumours from intracardiac injections appeared earlier compared with tumours from tail vein injections. (A) Tumour luminescence based on the number of days since the metastatic assay injection (intracardiac or tail vein). The assay injections (n = 15 for tail vein and n = 15 for intracardiac) were performed at separate times and the luminescence values were merged on one graph. (B) The number of tumours that developed following the metastatic assay injection (intracardiac or tail vein) based on the appearance of luminescent foci with signal persisting for more than one week.
**Fig. S3.** Quality control metrics for each cluster of cells within the RCC243 dataset. 0 represents the orthotopic tumour dataset, 1 represents the *in vitro* cell culture dataset, and 2 represents the metastatic tumour dataset. The metrics include the number of genes (nFeature_RNA) found in each cell, the number of unique molecular identifiers (nCount_RNA), and the percentage of mitochondrial genes expressed per cell (percent.mt).
**Fig. S4.** Top 20 differentially expressed genes based on each cluster of cells (0 represents orthotopic tumour dataset, 1 represents in vitro cell culture dataset, and 2 represents metastatic tumour dataset from tail vein or intracardiac injections).
**Fig. S5.** Gene set enrichment analysis between the orthotopic tumour cluster and the *in vitro* cell culture cluster reveals differential gene signatures. The hallmark pathways were from the Molecular Signatures Database. The most upregulated pathways for each cluster in this comparison are shown (orthotopic cluster at the top and in vitro cluster at the bottom).
**Fig. S6.** Gene set enrichment analysis between the orthotopic tumour cluster and the metastatic tumour cluster reveals differential gene signatures. The hallmark pathways were from the Molecular Signatures Database. The most upregulated pathways for each cluster in this comparison are shown (orthotopic cluster at the top and metastatic cluster at the bottom).
**Fig. S7.** Differential expression between the RCC243 tumour models and the ccRCC patient datasets. (A) Clustering of the datasets based on the whether the cell was from the RCC243 tumour models or from the ccRCC patient datasets without batch correction. (B) Batch correction performed using Harmony (max_iter = 1) reveals that the ccRCC patient datasets cluster together separately from the RCC243 tumour models. (C) Differential expression analysis showing the top 10 differentially expressed genes from the RCC243 tumour models or the ccRCC patient datasets.
**Fig. S8.** Quality control metrics for each cluster of cells within the RCC243 tumour model and ccRCC patient tumour datasets. The metrics include the number of genes (nFeature_RNA) found in each cell, the number of unique molecular identifiers (nCount_RNA), and the percentage of mitochondrial genes expressed per cell (percent.mt).
**Fig. S9.** Areas of low CA9 expression in a ccRCC tumour section are associated with a higher expression of FN1 compared with areas of high CA9 expression. Based on the tumour section in Fig. 4D from the Meylan, M. et al. dataset. The analysis was performed using the Loupe Browser 8.1.2 after the conversion of the Seurat file using LoupeR. Afterwards, the dataset was subsetted based on CA9 expression using LogNorm for scaling on the Loupe Browser. High CA9 expression was categorized by an expression value greater than 2 (62 areas) and low CA9 expression was categorized by an expression value lower than 0.5 (46 areas).
**Fig. S10.** Shannon entropy calculations of the RCC243 experimental models reveal an increased transcriptional heterogeneity within the orthotopic tumours compared with the metastatic tumours and in vitro cell culture models. The calculations were performed using the scbp package with an included function that uses Shannon entropy to evaluate the diversity of the clusters.
**Fig. S11.** Areas with high FN1 expression are associated with an increased expression of some markers of fibroblasts. The analysis was performed using the Loupe Browser 8.1.2 to perform the analysis following the conversion of the Seurat file using LoupeR. The dataset was categorized using a LogNorm value of greater than 3 to denote high FN1 expression and a value greater than 2 to indicate high CA9 expression (which is associated with a lower expression of FN1). Fibroblast markers such as COL1A1, COL1A2, LGALS1, SERPINE1, and VIM are more highly expressed in areas with higher FN1 expression.

**Table S1.** Excel file containing the gene list generated using MAST (orthotopic versus *in vitro* cell culture comparison) for Fig. 3A.

**Table S2.** Excel file containing the gene list generated using MAST (orthotopic tumor versus metastatic tumor comparison) for Fig. 3B.

**Table S3.** Excel file containing the gene list generated using MAST (RCC243 *in vivo* tumor model versus ccRCC patient tumor comparison).

**File S1.** Code for the RCC243 tumor model dataset analysis.
**File S2.** Code for the RCC243 tumor model and ccRCC patient tumor dataset analysis.
**File S3.** Code for the spatial transcriptomics ccRCC tumor analysis.

## Data Availability

The single‐cell RNA‐sequencing datasets generated in this study are publicly available in the Gene Expression Omnibus (GEO) at GSE272610. In addition, the single‐cell RNA‐sequencing and spatial transcriptomics datasets analyzed in this study were also obtained from the GEO at GSE188635, GSE152938, and GSE175540, as well as the European Genome‐Phenome Archive (EGAD00001004304) with approval from the Wellcome Trust Sanger Institute [23, 48, 55, 56].
