# Supplementary material for: Comparative single‐cell transcriptomic profiling of patient‐derived renal carcinoma cells in cellular and animal models of kidney cancer
Source: FEBS Open Bio. 2025 Apr 16;15(7):1124–43. doi: 10.1002/2211-5463.70022 (PMC12226427; doi:10.1002/2211-5463.70022)
Supplement: Supplementary file 1 — Fig. S1. Creation of the RCC243‐tdTomato cell line and confirmation of the depletion of mouse cells for single‐cell RNA‐sequencing. (A) A comparison of a healthy mouse kidney and a kidney affected by the injection of RCC243. (B) The confirmation of the generated RCC243‐tdTomato cell line by fluorescence activated cell sorting. (C) Confirmation of the depletion of mouse cells that were stained with the PE‐Cyanine7 fluorochrome following the tumour dissociation process for each model of RCC243. The analysis for the RCC243‐tdTomato intracardiac tumour sample may have been contaminated with cells present within the lineage positive fraction that contained the depleted mouse cells as it was analyzed immediately prior to the lineage negative fraction. Fig. S2. Tumours from intracardiac injections appeared earlier compared with tumours from tail vein injections. (A) Tumour luminescence based on the number of days since the metastatic assay injection (intracardiac or tail vein). The assay injections (n = 15 for tail vein and n = 15 for intracardiac) were performed at separate times and the luminescence values were merged on one graph. (B) The number of tumours that developed following the metastatic assay injection (intracardiac or tail vein) based on the appearance of luminescent foci with signal persisting for more than one week. Fig. S3. Quality control metrics for each cluster of cells within the RCC243 dataset. 0 represents the orthotopic tumour dataset, 1 represents the in vitro cell culture dataset, and 2 represents the metastatic tumour dataset. The metrics include the number of genes (nFeature_RNA) found in each cell, the number of unique molecular identifiers (nCount_RNA), and the percentage of mitochondrial genes expressed per cell (percent.mt). Fig. S4. Top 20 differentially expressed genes based on each cluster of cells (0 represents orthotopic tumour dataset, 1 represents in vitro cell culture dataset, and 2 represents metastatic tumour dataset from tail vein [file FEB4-15-1124-s004.docx]

**Supplementary Figures and Legends**

**Figure S1**

**
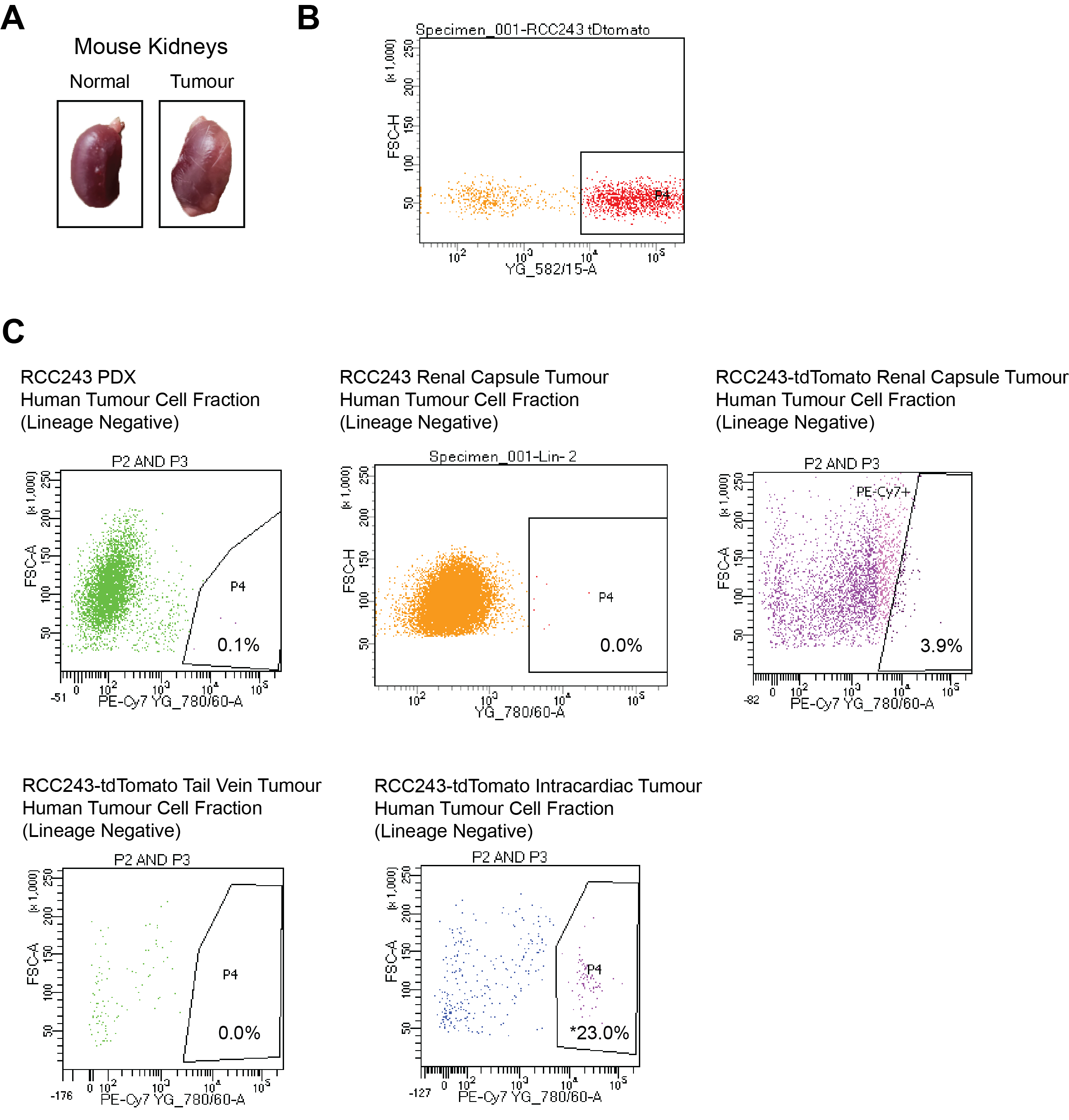
**

**Supplementary Figure 1. Creation of the RCC243-tdTomato cell line and confirmation of the depletion of mouse cells for single-cell RNA-sequencing.** A. A comparison of a healthy mouse kidney and a kidney affected by the injection of RCC243. B. The confirmation of the generated RCC243-tdTomato cell line by fluorescence activated cell sorting. C. Confirmation of the depletion of mouse cells that were stained with the PE-Cyanine7 fluorochrome following the tumour dissociation process for each model of RCC243. The analysis for the RCC243-tdTomato intracardiac tumour sample may have been contaminated with cells present within the lineage positive fraction that contained the depleted mouse cells as it was analyzed immediately prior to the lineage negative fraction.

**Figure S2**


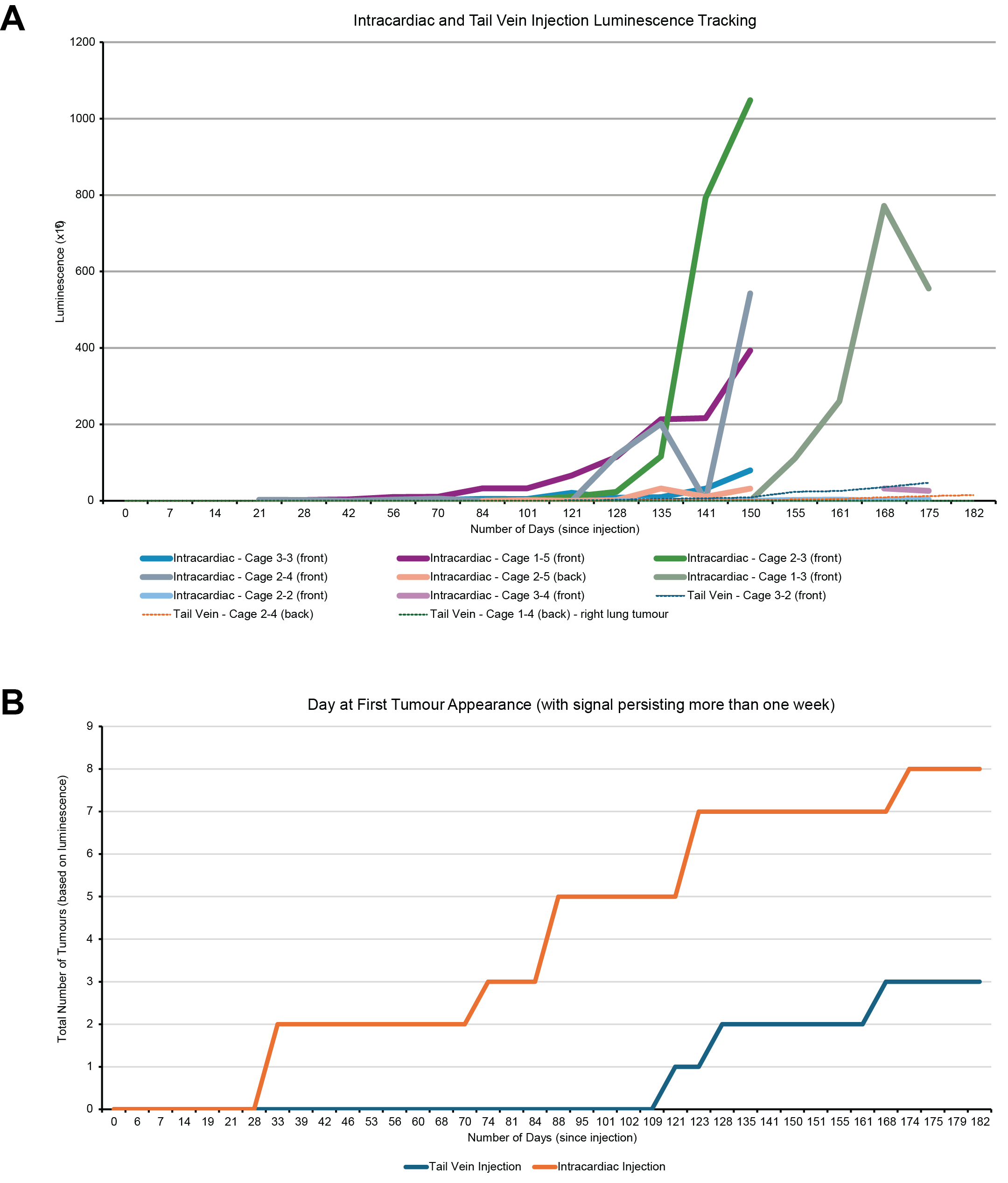


**Supplementary Figure 2. Tumours from intracardiac injections appeared earlier compared with tumours from tail vein injections.** A. Tumour luminescence based on the number of days since the metastatic assay injection (intracardiac or tail vein). The assay injections (n = 15 for tail vein and n = 15 for intracardiac) were performed at separate times and the luminescence values were merged on one graph. B. The number of tumours that developed following the metastatic assay injection (intracardiac or tail vein) based on the appearance of luminescent foci with signal persisting for more than one week.

**Figure S3**


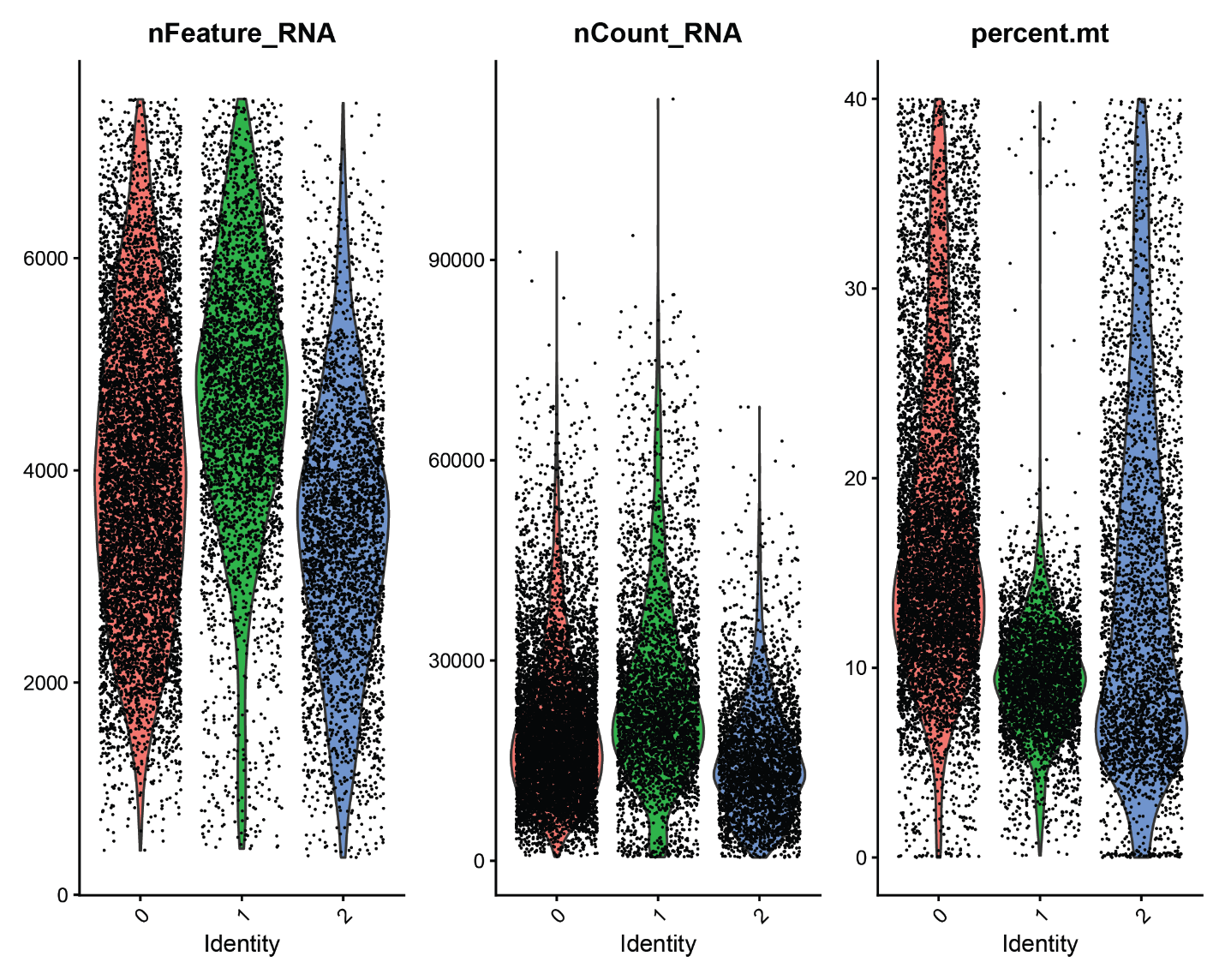


**Supplementary Figure 3. Quality control metrics for each cluster of cells within the RCC243 dataset.** 0 represents the orthotopic tumour dataset, 1 represents the *in vitro* cell culture dataset, and 2 represents the metastatic tumour dataset. The metrics include the number of genes (nFeature_RNA) found in each cell, the number of unique molecular identifiers (nCount_RNA), and the percentage of mitochondrial genes expressed per cell (percent.mt).

**Figure S4**

**
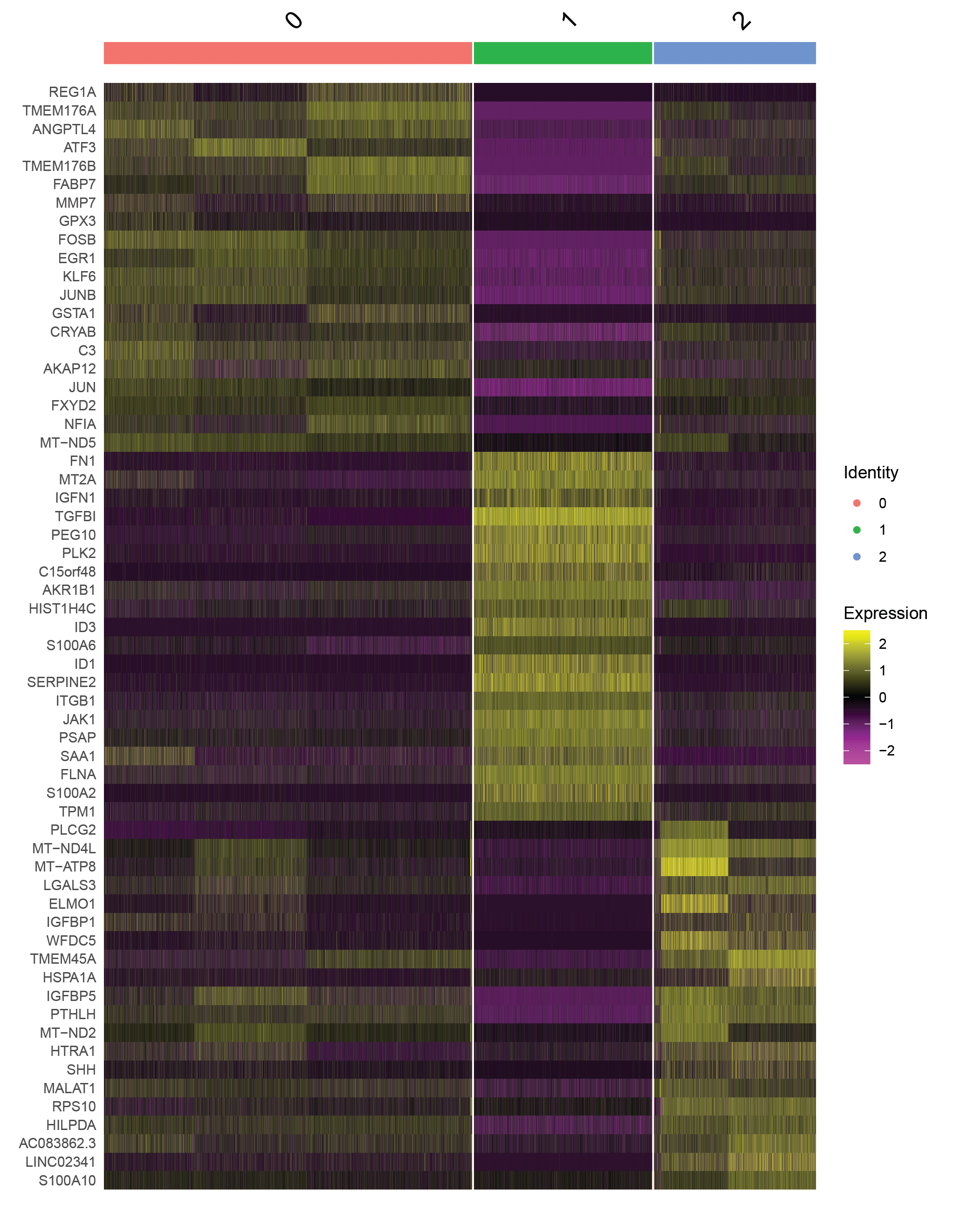
**

**Supplementary Figure 4. Top 20 differentially expressed genes based on each cluster of cells (0 represents orthotopic tumour dataset, 1 represents in vitro cell culture dataset, and 2 represents metastatic tumour dataset from tail vein or intracardiac injections).**

**Figure S5**


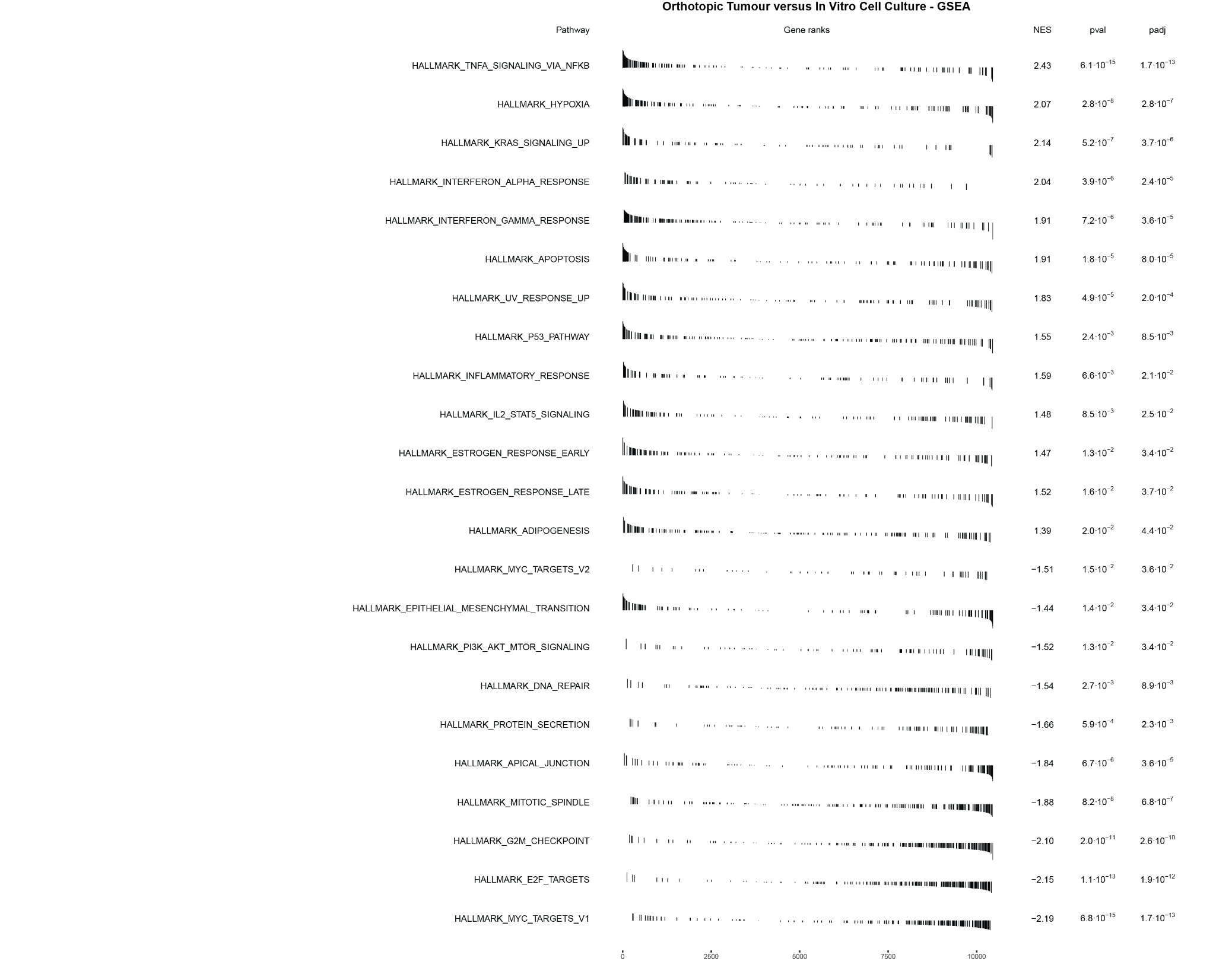


**Supplementary Figure 5. Gene set enrichment analysis between the orthotopic tumour cluster and the *in vitro* cell culture cluster reveals differential gene signatures.** The hallmark pathways were from the Molecular Signatures Database. The most upregulated pathways for each cluster in this comparison are shown (orthotopic cluster at the top and in vitro cluster at the bottom).

**Figure S6**


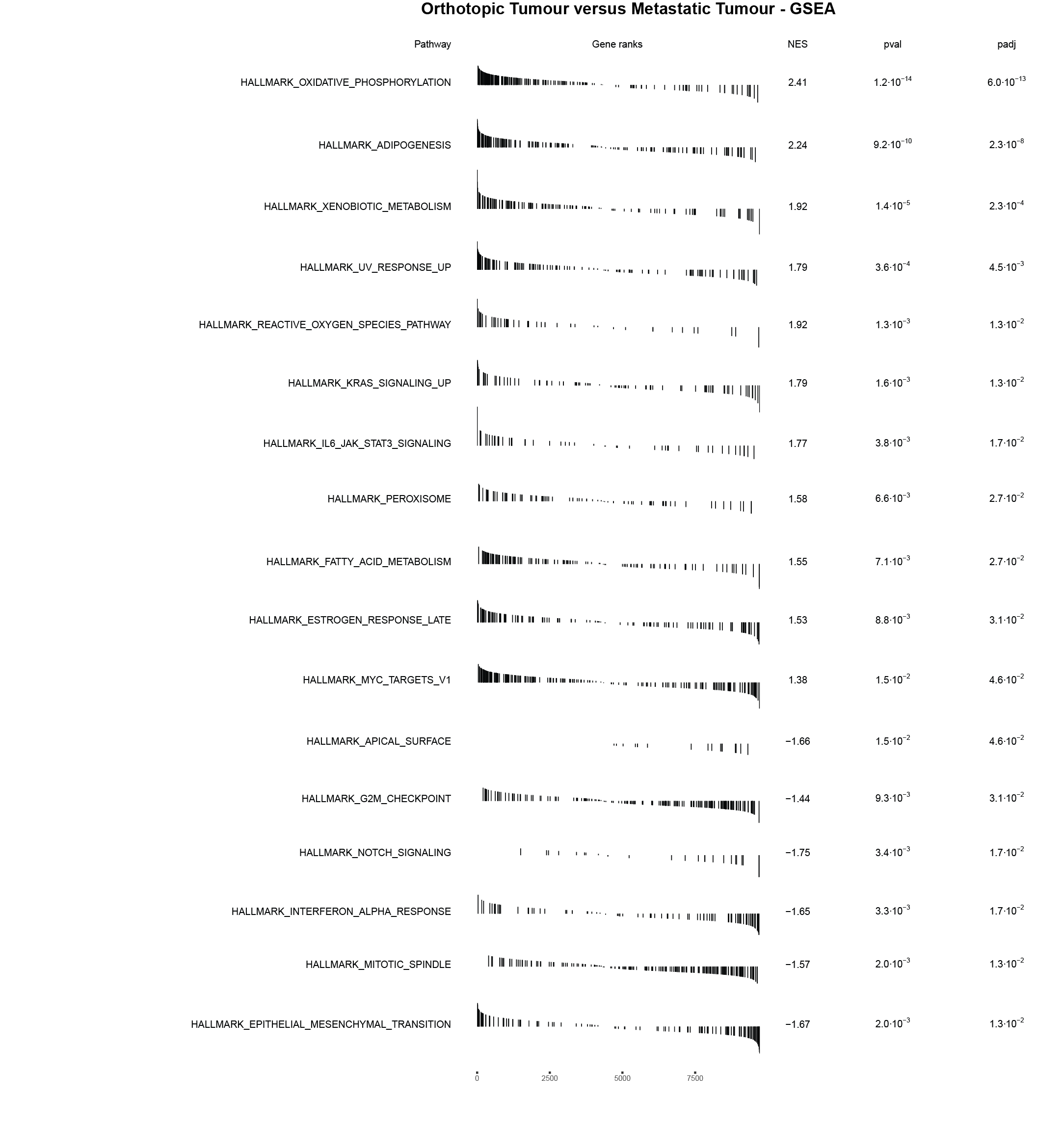


**Supplementary Figure 6. Gene set enrichment analysis between the orthotopic tumour cluster and the metastatic tumour cluster reveals differential gene signatures.** The hallmark pathways were from the Molecular Signatures Database. The most upregulated pathways for each cluster in this comparison are shown (orthotopic cluster at the top and metastatic cluster at the bottom).

**Figure S7**


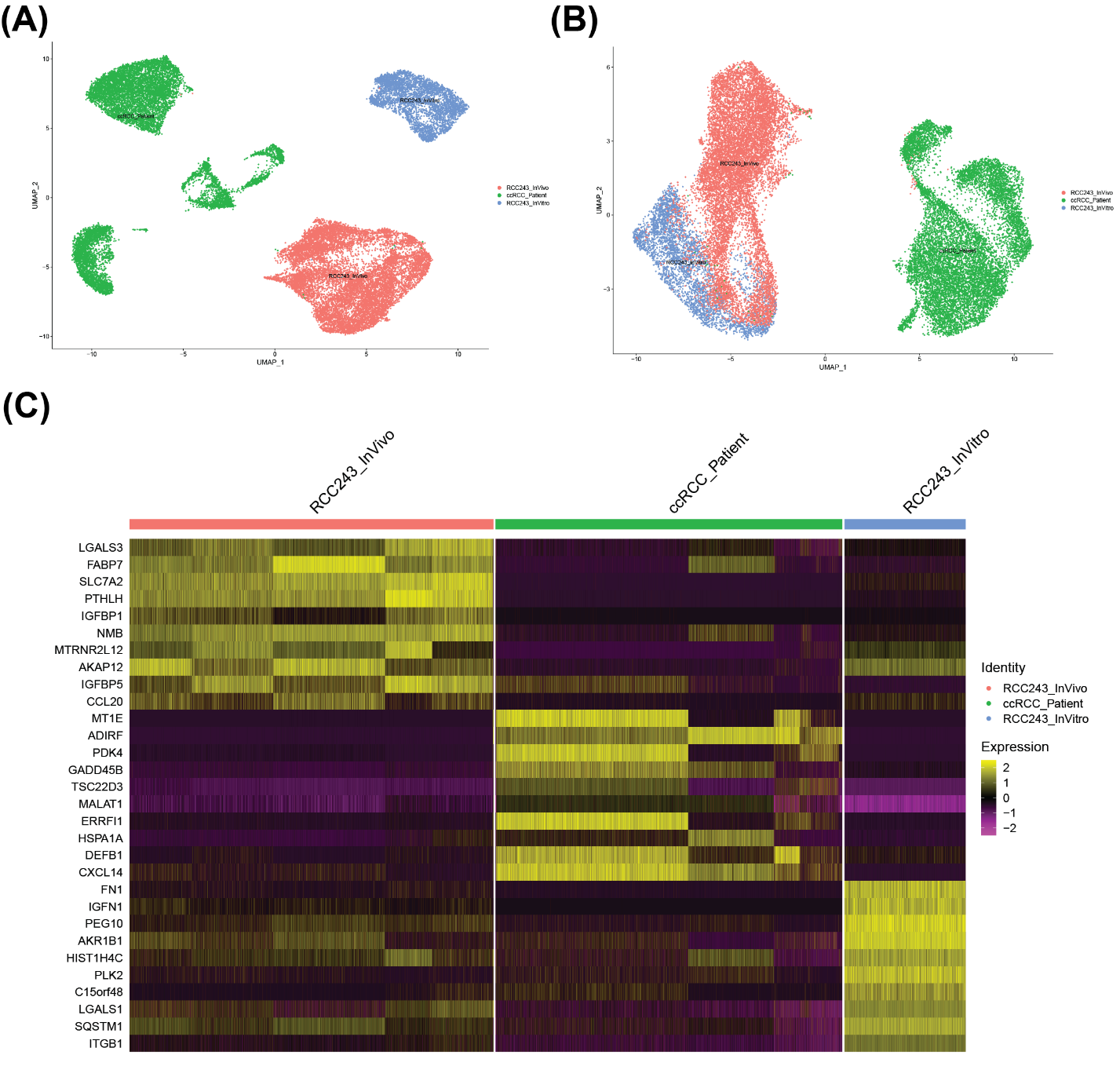


**Supplementary Figure 7. Differential expression between the RCC243 tumour models and the ccRCC patient datasets.** (A) Clustering of the datasets based on the whether the cell was from the RCC243 tumour models or from the ccRCC patient datasets without batch correction. (B) Batch correction performed using Harmony (max_iter = 1) reveals that the ccRCC patient datasets cluster together separately from the RCC243 tumour models. (C) Differential expression analysis showing the top 10 differentially expressed genes from the RCC243 tumour models or the ccRCC patient datasets.

**Figure S8**


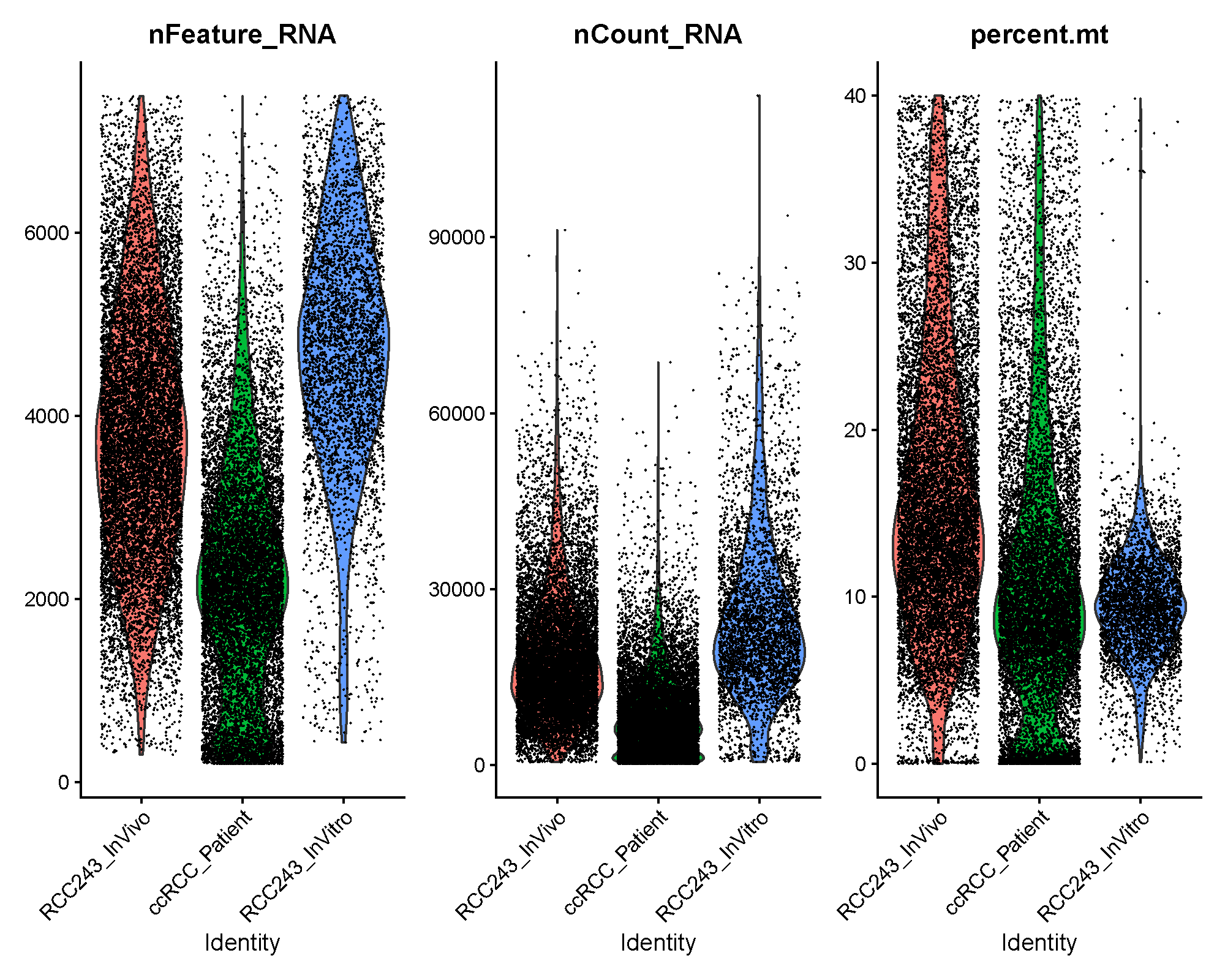


**Supplementary Figure 8. Quality control metrics for each cluster of cells within the RCC243 tumour model and ccRCC patient tumour datasets.** The metrics include the number of genes (nFeature_RNA) found in each cell, the number of unique molecular identifiers (nCount_RNA), and the percentage of mitochondrial genes expressed per cell (percent.mt).

**Figure S9**


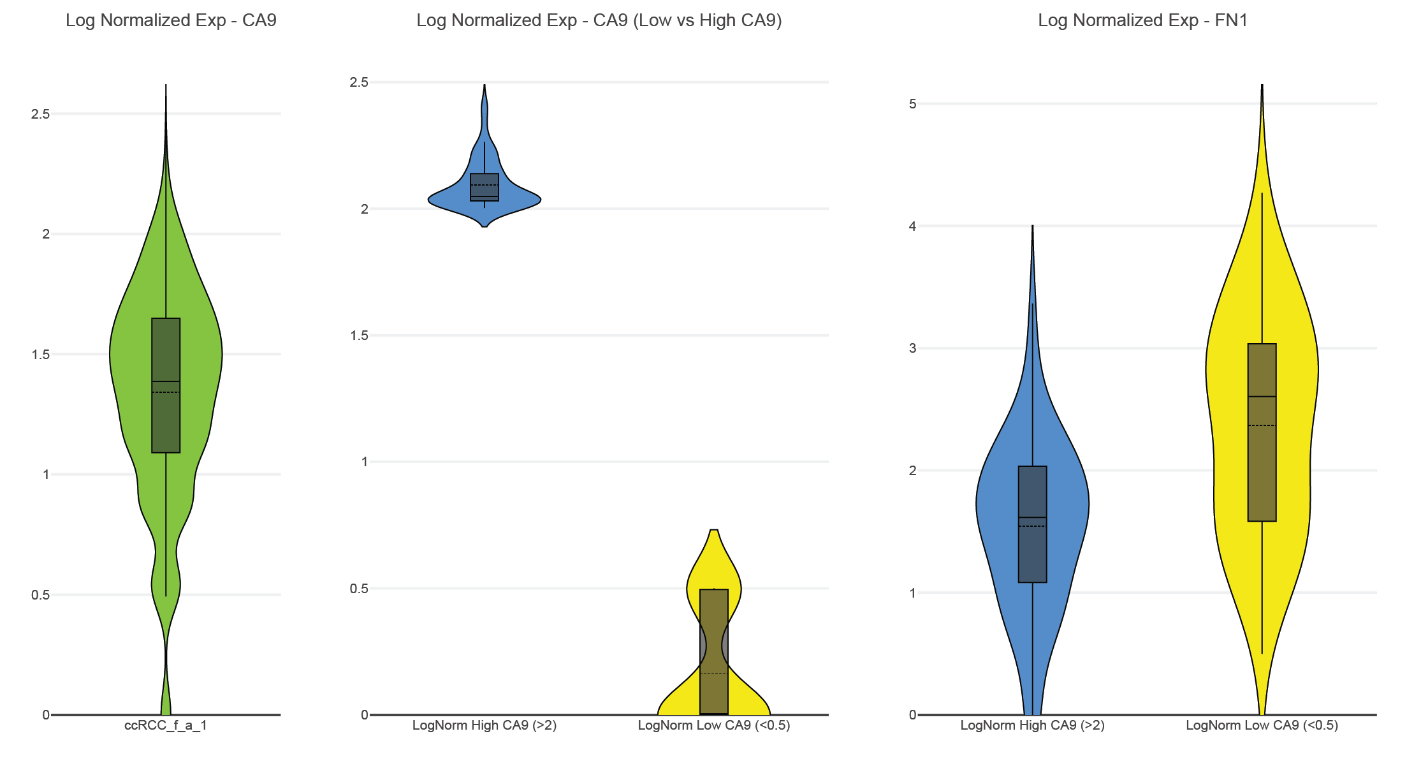


**Supplementary Figure 9. Areas of low CA9 expression in a ccRCC tumour section are associated with a higher expression of FN1 compared with areas of high CA9 expression.** Based on the tumour section in Fig. 4D from the Meylan, M. et al. dataset. The analysis was performed using the Loupe Browser 8.1.2 after the conversion of the Seurat file using LoupeR. Afterwards, the dataset was subsetted based on CA9 expression using LogNorm for scaling on the Loupe Browser. High CA9 expression was categorized by an expression value greater than 2 (62 areas) and low CA9 expression was categorized by an expression value lower than 0.5 (46 areas).

**Figure S10**

**
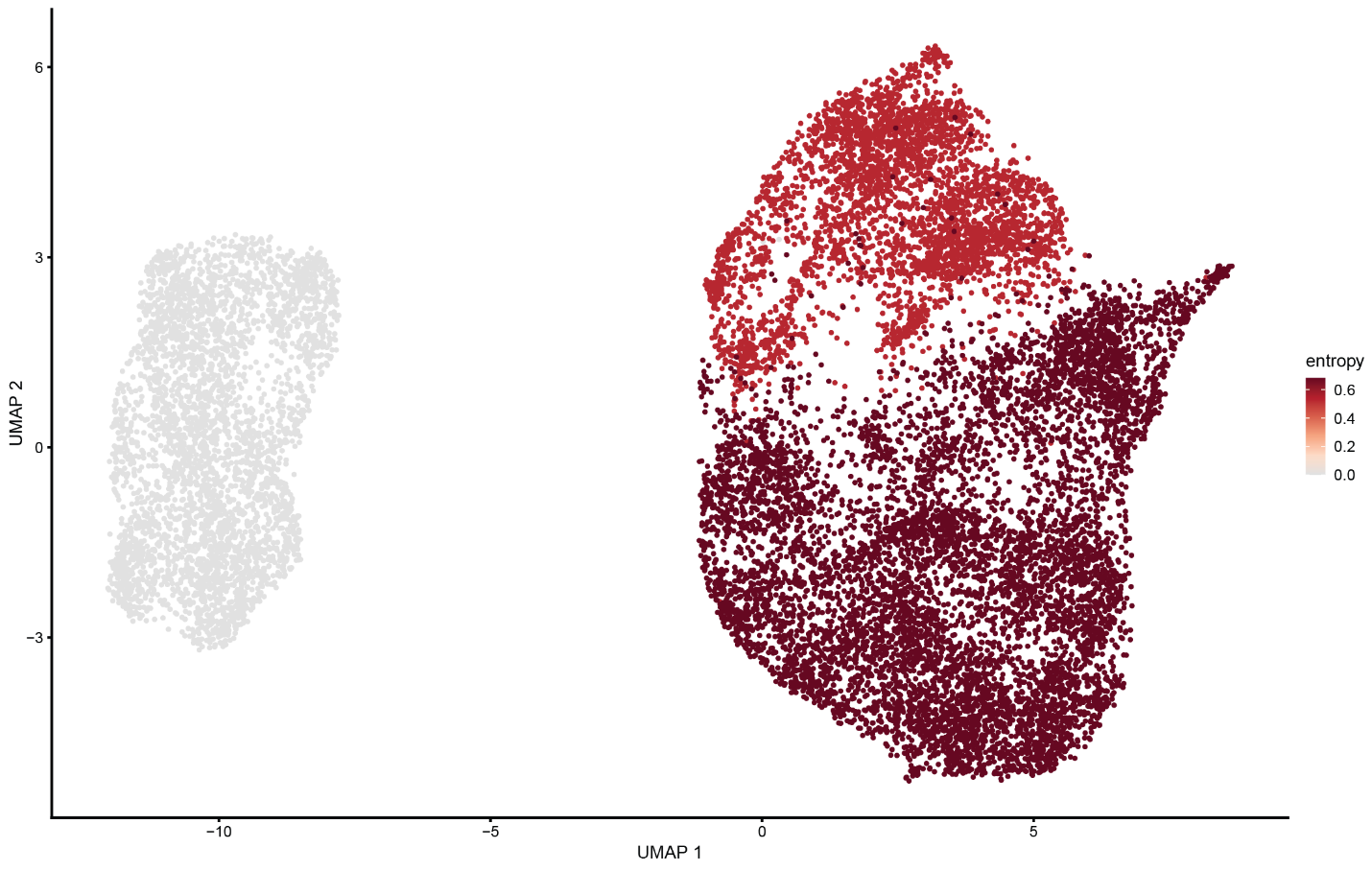
**

**Supplementary Figure 10. Shannon entropy calculations of the RCC243 experimental models reveal an increased transcriptional heterogeneity within the orthotopic tumours compared with the metastatic tumours and in vitro cell culture models.** The calculations were performed using the scbp package with an included function that uses Shannon entropy to evaluate the diversity of the clusters.

**Figure S11**


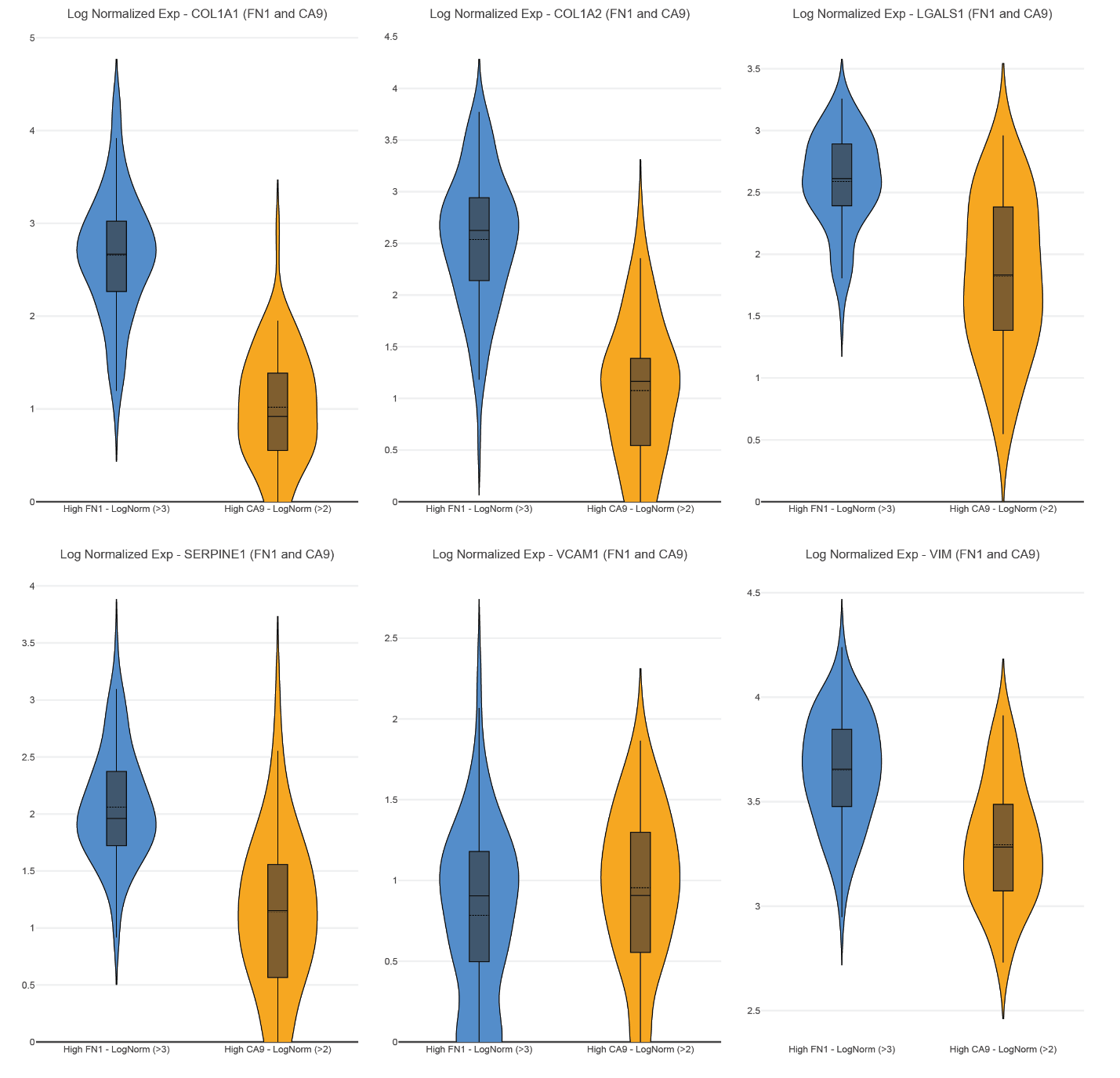


**Supplementary Figure 11. Areas with high FN1 expression are associated with an increased expression of some markers of fibroblasts.** The analysis was performed using the Loupe Browser 8.1.2 to perform the analysis following the conversion of the Seurat file using LoupeR. The dataset was categorized using a LogNorm value of greater than 3 to denote high FN1 expression and a value greater than 2 to indicate high CA9 expression (which is associated with a lower expression of FN1). Fibroblast markers such as COL1A1, COL1A2, LGALS1, SERPINE1, and VIM are more highly expressed in areas with higher FN1 expression.
